# N-acetylglucosamine sensing by a GCN5-related *N*-acetyltransferase induces transcription via chromatin histone acetylation in fungi

**DOI:** 10.1038/ncomms12916

**Published:** 2016-10-03

**Authors:** Chang Su, Yang Lu, Haoping Liu

**Affiliations:** 1 Department of Biological Chemistry, University of California, Irvine, California 92697, USA

## Abstract

N-acetylglucosamine (GlcNAc) exists ubiquitously as a component of the surface on a wide range of cells, from bacteria to humans. Many fungi are able to utilize environmental GlcNAc to support growth and induce cellular development, a property important for their survival in various host niches. However, how the GlcNAc signal is sensed and subsequently transduced is largely unknown. Here, we identify a gene that is essential for GlcNAc signalling (*NGS1*) in *Candida albicans*, a commensal and pathogenic yeast of humans. Ngs1 can bind GlcNAc through the N-terminal *β-N-*acetylglucosaminidase homology domain. This binding activates *N*-acetyltransferase activity in the C-terminal GCN5-related *N*-acetyltransferase domain, which is required for GlcNAc-induced promoter histone acetylation and transcription. Ngs1 is targeted to the promoters of GlcNAc-inducible genes constitutively by the transcription factor Rep1. Ngs1 is conserved in diverse fungi that have GlcNAc catabolic genes. Thus, fungi use Ngs1 as a GlcNAc-sensor and transducer for GlcNAc-induced transcription.

## Abstract (web summary)

Many fungi are able to metabolise environmental N-acetylglucosamine, however the mechanism by which this molecule is sensed is unclear. Su and Lu *et al*. show that *Candida albicans* NGS1 fulfils this function by mediating N-acetylglucosamine-dependent histone acetylation at target genes.

## Graphical abstract

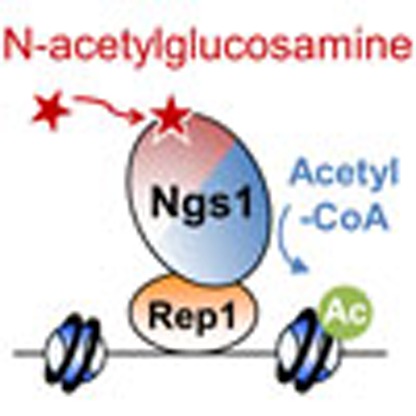

N-acetylglucosamine (GlcNAc) is a major structural component on the cell surface of a wide range of cells^1^. It is a component of the peptidoglycan of bacterial cell walls and the extracellular matrix glycosaminoglycans of animal cells. The polymer of GlcNAc, chitin, is a component of fungal cell walls and exoskeletons of arthropods such as crustaceans and insects. The GlcNAc released from these polymers is taken up and used by microbes. In bacteria, GlcNAc regulates the expression of GlcNAc synthesis and catabolic genes, virulence properties^1^, as well as cellular development and antibiotic production^2^. In fungi, GlcNAc induces GlcNAc catabolic genes and morphogenesis programs. Uptake and intracellular catabolism of GlcNAc have been mostly studied in *Candida albicans*, the major pathogenic yeast of humans. GlcNAc enters *C. albicans* cells by a plasma membrane transporter, Ngt1 (ref. 3). Intracellular GlcNAc induces the expression of three GlcNAc catabolic genes: GlcNAc kinase (Hxk1), GlcNAc-6-phosphate deacetylase (Dac1), and GlcN-6-phosphate deaminase (Nag1), all of which act sequentially to convert GlcNAc to fructose-6-phosphate which is then fed into the glycolytic pathway. The catabolic genes and *NGT1* are conserved in fungi^4^,^5^,^6^. Recently, a transcription factor was found to control the expression of GlcNAc catabolic genes in the filamentous fungus *Trichoderma reesei*^6^. Although some metabolites have been shown to activate gene expression through direct modulation of transcription factors in unicellular organisms^7^, little is known about the mechanism of how GlcNAc is sensed and how the signal is transduced to induce the expression of its catabolic genes in fungi. The lack of GlcNAc catabolic genes in *Saccharomyces cerevisiae* and *Schizosaccharomyces pombe* has hindered the discovery of a GlcNAc signalling pathway in fungi.

Chemical modifications of histones, such as methylation and acetylation, play critical roles in epigenetic gene regulation. Many of the enzymes that add or remove such chemical modifications are known to be sensitive to changes in intracellular metabolism. By neutralizing the positive charge of histone tails, acetylation serves to loosen histone-DNA interactions and allow transcription factors to access promoter chromatin^8^. Chromatin acetylation also provides binding sites for effector proteins to recruit coactivators for transcription^9^. Histone acetylation is established by the opposing functions of histone acetyltransferase (HAT) and deacetylase (HDAC) enzymes. Gcn5 was the first HAT that was discovered and extensively studied^10^. Structural studies of members of the superfamily of GCN5-related *N*-acetyltransferases (GNATs) provided the first atomic resolution structures of HATs for conserved mechanisms of acetyl-CoA interaction^11^. Enzymatic activities of HATs are regulated by phosphorylation, interaction with other proteins^10^, and the metabolic state of the cell^12^. However, the link between metabolic substrates and their ability to directly influence epigenetic signalling remains incompletely understood.

As a part of the commensal microbiota, *C. albicans* colonizes multiple mucosal sites, including the gastrointestinal (GI) tract. GlcNAc has been implicated in commensal growth of *C. albicans* in the GI tract^13^, where glucose is often limited and GlcNAc can be released from bacteria. *C. albicans* can also scavenge GlcNAc from glycosaminoglycan of the host cell extracellular matrix by secreting a GlcNAc-inducible β-hexosaminidase^14^. The ability to use environmental GlcNAc is important for the existence of *C. albicans* in its host niches, as GlcNAc catabolic pathway mutants have significantly reduced virulence^15^,^16^,^17^. Not only can GlcNAc induce genes for GlcNAc catabolism, it can also promote *C. albicans* cells to undergo hyphal development^18^ and phenotypic switching between two heritable cell states^19^. These morphogenesis programs of *C. albicans* are linked to its virulence, infection of distinct host niches and immune evasion^20^,^21^. In addition to *C. albicans*, GlcNAc also triggers a rapid morphogenetic programme in two thermally dimorphic fungal pathogens, *Histoplasma capsulatum* and *Blastomyces dermatitidis*^4^. Uncovering the mechanism for GlcNAc signalling is important for understanding fungal pathogenesis.

Here we report a gene that is required for N-acetylglucosamine signalling (*NGS1*) and catabolism. Ngs1 contains a conserved GlcNAc binding pocket at its N-terminus and a C-terminal GNAT domain; both are important for GlcNAc-induced promoter chromatin acetylation and transcription of GlcNAc catabolic genes. Ngs1 also controls other GlcNAc-induced transcription programs and is conserved in diverse fungi. Thus, Ngs1 is a novel GlcNAc signal sensor-transducer in fungi.

## Results

### Identifying a novel protein essential for GlcNAc signalling

**Figure 1:**
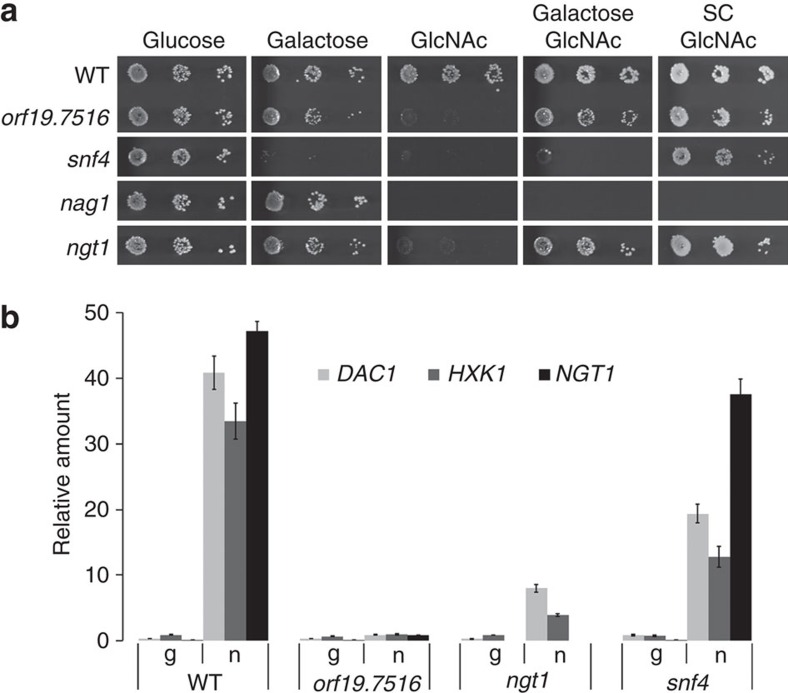
*ngs1* (*orf19.7516*) and *ngt1* are specifically defective in GlcNAc signalling and catabolism. (**a**) Cells of wild-type (SN250), *orf19.7516, snf4, nag1* and *ngt1* (HLY4394) mutant strains were serially diluted 10-fold and spotted onto YNB solid medium containing 2.5 mM of the indicated sugar or SC solid medium containing 2.5 mM GlcNAc. The YNB medium was supplemented with 0.1 mg ml^−1^ arginine to permit growth. Photographs were taken after 2 days of growth at 30 °C. (**b**) qRT-PCR analysis of GlcNAc catabolic genes *DAC1* and *HXK1* and GlcNAc transporter *NGT1* upon GlcNAc induction in the wild type (SN250) and indicated mutants. Cells were grown in liquid SC medium with 2.5 mM GlcNAc (n) or 5% glycerol (g) for 2 h at 30 °C for RNA extraction. Mean data±s.d. from three independent qRT-PCR experiments was plotted.

To uncover the GlcNAc signalling pathway, we performed a genetic screen with *C. albicans* deletion libraries for mutants defective in growth on GlcNAc as the sole carbon source. None of the 165 transcriptional factors^22^ and none of the 80 kinases and kinase-related genes^23^ is required for growth on GlcNAc. From a knockout library of 674 unique genes in *C. albicans*^24^, three mutants (*nag1, snf4, orf19.7516* (*CR_00190W*)) were found to be unable to grow on GlcNAc (Fig. 1a). orf19.7516 is specifically required for GlcNAc utilization as the *orf19.7516* mutant grew well on galactose or on GlcNAc media supplemented with galactose or amino acids, similar to the GlcNAc transporter mutant *ngt1* (ref. 3). Snf4 is not specific for GlcNAc signalling as it is also required for growth on galactose, consistent with its function in *S. cerevisiae* for metabolizing alternative carbon sources in the absence of glucose^25^. As reported, the *nag1* showed a growth defect on all GlcNAc containing media, even when supplemented with galactose or amino acids, due to GlcNAc-6-PO_4_ accumulation that is suggested to inhibit the growth of *C. albicans* cells^26^. We also screened the GRACE library, a non-redundant library containing a total of 2,357 different mutants^27^. *ngt1* and *orf19.7516* were the only two mutants found specifically defective in GlcNAc utilization from the GRACE library and the deletion libraries we screened, which cover about 40% genes in the genome.

To determine if the *orf19.7516* mutant is defective in GlcNAc signalling, we examined whether GlcNAc catabolic genes or the transporter *NGT1* are induced. *ORF19.7516* was essential for the induction of *HXK1, DAC1* and *NGT1* in response to GlcNAc (Fig. 1b). Therefore, we designate *ORF19.7516* as *NGS1* for N-acetylglucosamine signalling. Intracellular GlcNAc is responsible for GlcNAc signalling in *C albicans*^26^; however, we detected a slight increase in *HXK1* and *DAC1* expression in the *ngt1* mutant, which could be attributed to the existence of a low affinity GlcNAc uptake process^3^,^28^. The essential function of Ngs1 in GlcNAc signalling is not due to its requirement for *NGT1* expression and GlcNAc transport because ectopic expression of *NGT1* could not bypass the growth defect of the *ngs1* mutant on GlcNAc (Supplementary Fig. 1a). The growth defect of the *ngs1* mutant on GlcNAc could be rescued by reintroducing a copy of *NGS1* under its own promoter or an N-terminally FLAG-tagged Ngs1 (Supplementary Fig. 1a). Ngs1 levels were similar in cells grown in glucose or GlcNAc (Supplementary Fig. 2a,b), suggesting that GlcNAc signalling is not mediated by changing Ngs1 levels.

### GlcNAc binding with Ngs1 is essential for GlcNAc signalling

**Figure 2:**
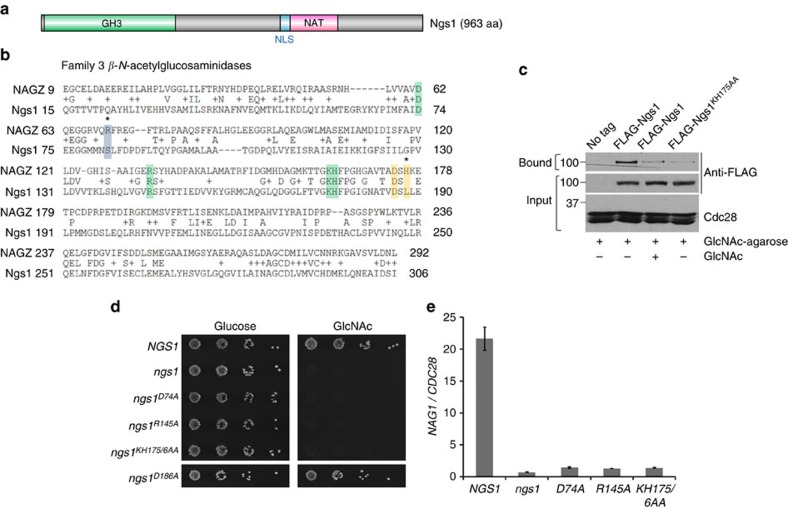
Binding of GlcNAc to Ngs1 is required for GlcNAc signalling. (**a**) Schematic depiction of the Glycoside Hydrolase family 3 (GH3) domain (green box), *N-*Acyltransferase (NAT) domain (pink box), and nuclear localization signal (NLS) region (blue box) in Ngs1. (**b**) Sequence alignment of the GH3 *β-N-*acetylglucosaminidase domain between Ngs1 and *St*NagZ. The residues known to interact with GlcNAc (boxed in green) or anhMurNAc (boxed in blue), and the Asp-His dyad involved in catalysis (boxed in yellow) are indicated. Residues divergent in Ngs1 are indicated by asterisks. (**c**) Binding of Ngs1 to GlcNAc-agarose beads. Protein extracts from the *ngs1* mutants expressing untagged Ngs1 (HLY4395), FLAG-tagged Ngs1 (HLY4402), or FLAG-tagged Ngs1^KH175/6AA^ (HLY4405) were incubated with GlcNAc-agarose at 4 °C overnight in the absence or presence of 100 mM GlcNAc. The input and bound fractions were analysed on Western blots probed with FLAG antibody. (**d**) Mutations in the GlcNAc binding sites of Ngs1 abolished growth on GlcNAc. *ngs1* mutant cells carrying a wild-type copy of *NGS1* (HLY4395), *ngs1*^*D74A*^ (HLY4398), *ngs1*^*R145A*^ (HLY4399), *ngs1*^*KH175/6AA*^ (HLY4400), *ngs1*^*D186A*^ (HLY4401) or vector alone (HLY4396) were serially diluted 10-fold and spotted onto YNB solid medium containing 2.5 mM GlcNAc or glucose. Colony growth was assessed after 2 days of growth at 30 °C. (**e**) GlcNAc could not induce *NAG1* expression in strains carrying mutations in GlcNAc binding sites in Ngs1. Cells were grown in liquid SC medium with 2.5 mM GlcNAc for 2 h at 30 °C for RNA extraction. All data showed the average of three independent qRT-PCR experiments with error bars representing the s.d.

*NGS1* encodes an uncharacterized protein of 963 amino acids. The N-terminus of Ngs1 contains high conservation to *β-N-*acetylglucosaminidases in the Glycoside Hydrolase family 3 (GH3)^29^, which exhibit unique substrate specificity for GlcNAc^30^ (Fig. 2a,b). The C-terminal region of Ngs1 has a weak homology to the *N*-Acyltransferase (NAT) domain in the GCN5-related *N*-acetyltransferase (GNAT) family^31^,^32^ (Fig. 2a). In front of the NAT domain lays a putative nuclear localization signal (NLS), predicted by cNLS Mapper^33^ (Fig. 2a). The two putative, functionally distinct domains of Ngs1 are both required for Ngs1 function, as expressing the N-terminus (aa 1–341) or C terminus (aa 341–963) of Ngs1 was insufficient to complement the growth defect of *ngs1* on GlcNAc (Supplementary Figs 1a and 3a).

A pivotal question is whether the intracellular GlcNAc signal is transmitted via the GH3 *β-N-*acetylglucosaminidase homology domain. The N-terminal region of *Ngs1* (aa 15–306) is most similar to bacterial NagZs with 28% identity to *Salmonella typhimurium* NagZ (Fig. 2b). The GH3 NagZ enzymes participate in bacterial cell wall recycling by removing terminal GlcNAc from GlcNAc-anhMurNAc-peptide in Gram-negative bacteria^34^ and GlcNAc-MurNAc-peptide in Gram-positive bacteria^35^. Crystal structure determinations of NagZ enzymes in complex with their natural substrates (for example, GlcNAc-anhMurNAc) and its product GlcNAc have defined binding coordinates/residues of the NagZ family of enzymes for GlcNAc and anhMurNAc^36^. The conserved key GlcNAc binding sites in *St*NagZ are also present in Ngs1 (Fig. 2b, green shade), whereas the binding site for the lactyl moiety of anhMurNAc is divergent in Ngs1 (Fig. 2b, blue shade)^36^, which suggest that Ngs1 has GlcNAc monomer binding sites, but has lost the binding pocket for the disaccharide GlcNAc-anhMurNAc. In support of the sequence conservation, agarose conjugated with GlcNAc was able to pull down FLAG-Ngs1 (Fig. 2c). The binding of GlcNAc-agarose with FLAG-Ngs1 was greatly reduced when free GlcNAc was added to the cell lysate before incubation with GlcNAc-agarose. The interaction was also disrupted by mutating the conserved GlcNAc binding residues K175H176 in the GH3 domain of Ngs1. Thus, Ngs1 can bind GlcNAc via the conserved GlcNAc binding pocket. We further showed that the GlcNAc binding sites in Ngs1 are critical for its function in GlcNAc signalling, as mutations in the conserved residues D74, R145 and K175H176 resulted in a dramatic growth defect on GlcNAc (Fig. 2d). Addition of GlcNAc could not induce the expression of catabolic genes in these mutants (Fig. 2e). Mutating the GlcNAc binding sites did not affect their expression (Supplementary Fig. 3b). Altogether, our data suggested that Ngs1 can bind GlcNAc, and this binding is important for its function of sensing intracellular GlcNAc. Ngs1 is able to sense GlcNAc at micromolar levels and induce GlcNAc catabolic genes within minutes (Supplementary Fig. 4).

Since Ngs1 is highly homologous to GH3 *β-N-*acetylglucosaminidase, one obvious question is whether Ngs1 is a hydrolase. Most NagZ enzymes from Gram-negative bacteria are single domain enzymes and their catalytic domain contains the conserved aspartate nucleophile and a catalytic histidine/aspartate dyad^29^,^36^ (Fig. 2b, shaded in yellow). The conserved Asp-His dyad of NagZs are essential for the catalytic reaction of this family of enzymes. However, the histidine in the Asp-His dyad of NagZ is replaced by a leucine in Ngs1. Also, replacement of the Asp186 with alanine did not affect Ngs1′s function (Fig. 2d). Therefore, we suggest that Ngs1 is not a hydrolase. Consistent with this notion, we could not detect *N*-acetylglucosaminidase activity in an affinity pull-down of FLAG-Ngs1 from *C. albicans* cells grown in GlcNAc. Thus, the GlcNAc binding ability, but not hydrolase activity, is important for Ngs1's function in GlcNAc signalling.

### Ngs1 is associated with promoters of GlcNAc catabolic genes

**Figure 3:**
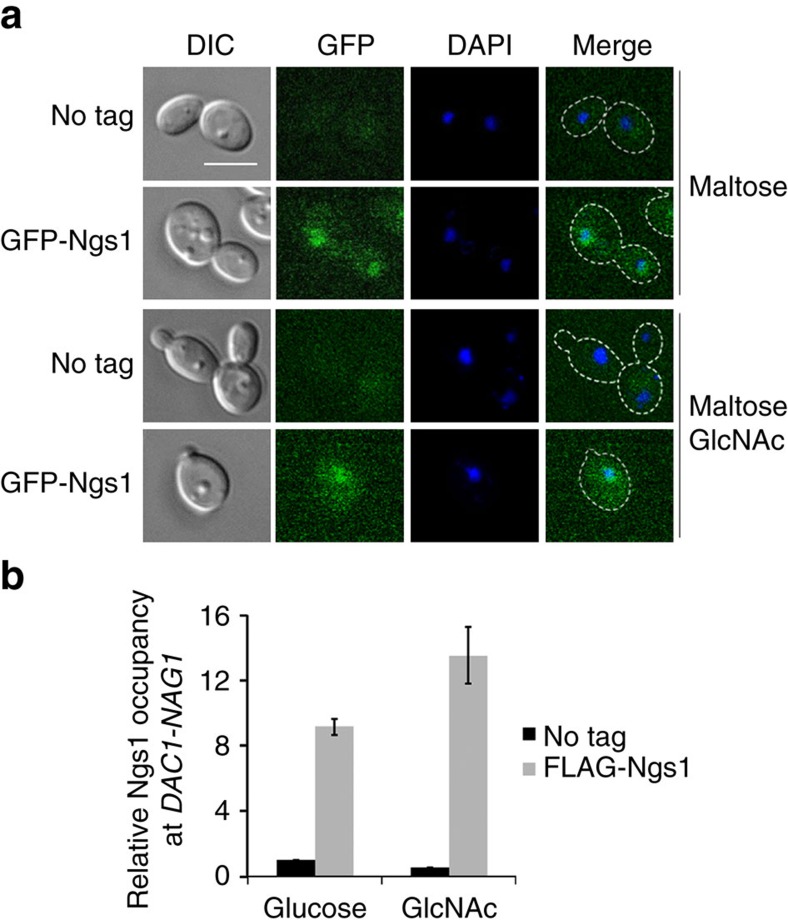
Ngs1 binds to the promoter of GlcNAc catabolic genes. (**a**) The *ngs1* mutant strain expressing GFP-Ngs1 under the *MAL2* promoter (HLY4397) was grown to log phase in liquid SC medium containing 50 mM maltose at 30 °C, and then treated with or without 50 mM GlcNAc for 30 min. Cell morphology and Ngs1 localization were observed by using DIC and fluorescence microscopy. An untagged control (SC5314) was included. For all images: Scale bar, 5 μm. (**b**) Ngs1 is present at the promoter for *NAG1* and *DAC1* in a GlcNAc-independent manner. Overnight cultures of *ngs1* mutant cells carrying 3FLAG-Ngs1 (HLY4402) or untagged Ngs1 (HLY4395) from the *NGS1* promoter were pelleted, washed three times in PBS, diluted 1:20 in SC medium with 10 mM GlcNAc or glucose at 30 °C, and cells were collected at 30 min for the ChIP experiment. ChIP DNA was quantitated as described^64^ by qPCR with primers at the promoter region of *NAG1* and *DAC1*. The *ACT1* promoter region was used as a control. The enrichment is presented as a ratio of *NAG1-DAC1* promoter IP (bound/input) versus *ACT1* promoter IP (bound/input). The value in *ngs1* mutant cells carrying untagged Ngs1 (HLY4395) in glucose medium was set to 1.00. The ChIP data showed the average of three independent qPCR data with error bars representing the s.d.

The rapid response to GlcNAc indicates that regulators in the GlcNAc signalling pathway are preexisting. Indeed, Ngs1 protein levels are unchanged before or after GlcNAc induction (Supplementary Fig. 2a). Functional GFP-Ngs1 expressed from the *MAL2* promoter (Supplementary Fig. 5) accumulated in the nucleus in the absence or presence of GlcNAc (Fig. 3a). To determine if Ngs1 is present at the promoters of its target genes, functional N-terminal FLAG-tagged Ngs1 was used to determine its association at the divergent promoter region shared by *NAG1* and *DAC1* by chromatin immunoprecipitation. Ngs1 was found associated with the promoter in a GlcNAc-independent manner (Fig. 3b). Therefore, Ngs1 functions at the promoter of GlcNAc catabolic genes to activate transcription in response to GlcNAc.

### The HAT activity in Ngs1 GNAT domain

**Figure 4:**
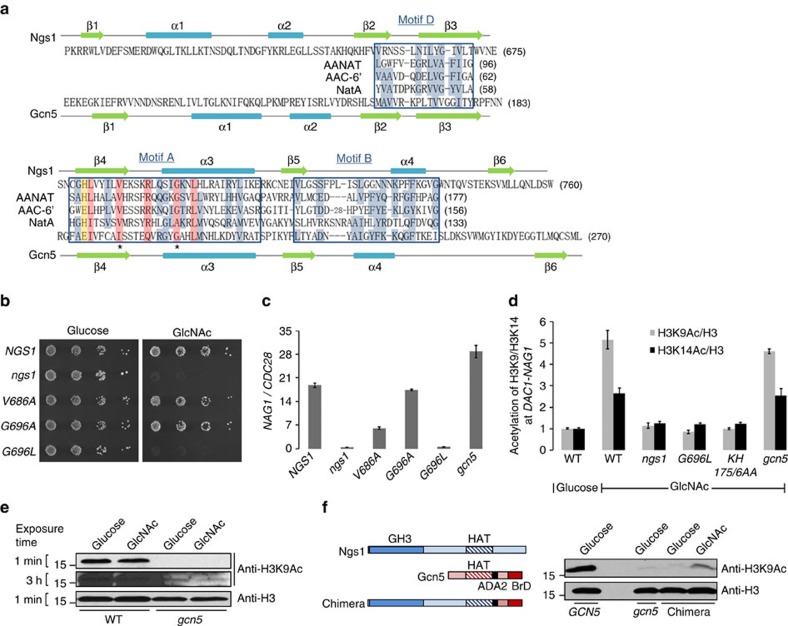
Conserved acetyl-CoA interacting residues of Ngs1 are required for GlcNAc-induced promoter histone acetylation and transcription. (**a**) Sequence alignment of GNAT homology motifs A, B, and D in Ngs1 and *C. albicans* Gcn5 and three GNAT superfamily members (sheep AANAT, arylalkylamine-*N*-acetyltransferase^68^; *Enterococcus faecium* AAC-6′, aminoglycoside 6′-*N*-acetyltransferase^32^; *S. pombe* NatA, *N*-acetyltransferase complex catalytic subunit^57^). Consensus residues throughout the superfamily, as determined by Neuwald and Landsman^40^, are shown with blue shade, and residues particularly conserved in motif A are shaded in pink. The yellow shade indicates the residue known to be critical for HAT catalysis in *Tetrahymena* Gcn5. The asterisks under the aligned sequences show the location of residues known to interact with the Acetyl-CoA. Secondary structure elements of Ngs1 and Gcn5 from *C. albicans* are also shown. (**b**) Dilutions of *ngs1* mutant cells carrying *NGS1* (HLY4395), *ngs1*^*V686A*^ (HLY4406), *ngs1*^*G696A*^ (HLY4407), *ngs1*^*G696L*^ (HLY4408), or vector alone (HLY4396) were spotted onto the solid YNB media plates containing 2.5 mM GlcNAc or glucose and then incubated at 30 °C for 2 days. (**c**) *NAG1* mRNA levels of the strains from (**b**) and the *gcn5* mutant (CPS50) were determined in GlcNAc-containing medium by qRT-PCR. (**d**) ChIP with anti-H3, anti-acetylated H3K9, and anti-acetylated H3K14 antibodies in the wild type (SN250), *ngs1* mutant cells carrying *ngs1*^*G696L*^ (HLY4408), *ngs1*^*KH175/6AA*^ (HLY4400) or vector alone (HLY4396), and *gcn5* mutant cells carrying vector (CPS50). Cells were grown in GlcNAc or glucose for 30 min for the ChIP experiment. The value from wild type in glucose medium was set to 1.00. (**e**) Global H3K9 acetylation level is not induced by GlcNAc. Cell lysates of wild type (SN250) and *gcn5* mutant (CPS50) growing in GlcNAc or glucose medium were subjected to Western analysis. (**f**) GlcNAc regulated H3K9 acetylation by the Ngs1-Gcn5 chimera in the *gcn5* mutant. The *gcn5* revertant and *gcn5* mutant carrying the empty vector or chimeric construct were grown overnight in YEP containing 100 mM GlcNAc or glucose at 30 °C to saturation for Western analysis. The construct is schematically illustrated on the left (ADA2, ADA2 interaction domain; BrD, bromodomain). Mean data±s.d. from three independent qPCR experiments was plotted.

The C-terminal region of Ngs1 is essential for growth on GlcNAc and contains a weak sequence homology to the NAT domain of the GCN5-related *N*-acetyltransferase (GNAT) family^31^,^32^. GNAT members are known to share weak similarities by sequence alignment, but they all have a universally conserved secondary structure^37^, containing a fold comprised of an N-terminal strand followed by two helixes, three β strands, followed by a signature central helix, a fifth β strand, a fourth α helix and a final β strand. The secondary structure is also found in the C-terminal region of Ngs1 using Jpred^38^ and PredictProtein^39^ (Fig. 4a). Members of the GNAT superfamily usually have four conserved motifs A to D^40^. Motif A is the core of the GNAT domain and is conserved in all members. Motif C is missing in some GNATs^9^. Ngs1 contains motifs A, B and D (Fig. 4a). The highly conserved residues in motif A are also conserved in Ngs1 (Fig. 4a)^32^. The conserved catalytic residue can be either glutamate or histidine (Fig. 4a, shaded in yellow)^31^,^41^.

To determine if the predicted *N*-acetyltransferase activity in Ngs1 is required for GlcNAc signalling, point mutations were introduced to the residues that are known to interact with Acetyl-CoA in GNATs (Fig. 4a, asterisk mark)^32^. Val686 and Gly696 were substituted with alanine. We also mutated Gly696 to leucine because several GNATs have alanine instead of glycine at this position^42^. The function of each mutant Ngs1 was tested in the *ngs1* mutant. The *ngs1*^*G696L*^ mutant could not grow on GlcNAc like the *ngs1* deletion mutant (Fig. 4b), and was unable to express *NAG1* in response to GlcNAc (Fig. 4c). In addition, cells carrying the V686A mutation showed impaired induction of *NAG1*. The protein levels of both *ngs1*^*G696L*^ and *ngs1*^*V686A*^ are largely unchanged compared to that of wild-type Ngs1 (Supplementary Fig. 3c). The G696A mutaion had no detectable phenotype as expected. Thus, the conserved acetyl-CoA interacting residues in the predicted *N*-acetyltransferase domain of Ngs1 are essential for GlcNAc signalling.

GNATs catalyse the transfer of an acetyl group from Acetyl-CoA to the amine of a wide range of substrates, including small molecules and proteins. For example, AANAT, NatA and AAC-6′ acetylate arylakylamine, protein N-terminal or aminoglucoside, respectively. The founding member Gcn5 functions as a coactivator that facilitates initiation of transcription by acetylating N-terminal lysines on H3 (ref. 10). We reasoned that this could be a possible mechanism for how promoter-associated Ngs1 regulates transcription upon GlcNAc induction. By immunoprecipitation with anti-acetylated-H3K9 antibodies, we observed a dramatic increase in the level of acetylated H3K9 at the promoter region of *DAC1* and *NAG1* in wild-type cells within 30 min of GlcNAc induction (Fig. 4d), in agreement with the role of H3K9ac in transcriptional initiation^43^,^44^. An increase in the level of H3K14ac was also observed upon addition of GlcNAc, albeit the fold of increase was lower than that of H3K9ac. Importantly, the GlcNAc-induced increase in H3K9 and H3K14 acetylation was abolished in the *ngs1* deletion mutant or *ngs1*^*G696L*^ mutant (Fig. 4d), suggesting an essential role of the *N*-acetyltransferase domain of Ngs1 for its function in histone acetylation in response to GlcNAc.

The *C. albicans* Gcn5, a homologue of *S. cerevisiae* Gcn5, is a major HAT for histone H3 acetylation in *C. albicans*^45^. Consistent with this notion, we found that acetylation at H3K9 is much lower in the *gcn5* mutant (Fig. 4e). Despite the major role Gcn5 plays in H3K9ac, it is not required for GlcNAc-induced chromatin acetylation of H3K9 and H3K14 or transcription (Fig. 4c,d). Therefore, the GlcNAc-induced increase in chromatin H3 acetylation is mediated through the C-terminal GNAT domain of Ngs1 but not Gcn5. However, we cannot detect any increase in the total level of H3K9ac upon GlcNAc induction even in *gcn5* mutant cells where the basal level of H3K9ac is low (Fig. 4e). ScGcn5 cannot acetylate nucleosomal histones by itself^10^; its substrate preference is expanded in the context of HAT complexes (Ada and SAGA). Thus the difference of substrate specificity between Gcn5 and Ngs1 is, at least in part, modulated by other proteins within the HAT complexes. ScGcn5 contains a roughly 160-residue HAT domain and an adjacent C-terminal domain that contains an ADA2-interaction domain and a conserved bromodomain, which may provide contact sites for other proteins within the HAT complexes. In order to determine if the GNAT domain in Ngs1 has the predicted HAT activity in response to GlcNAc, the GNAT domain of Gcn5 was replaced by the N-terminus of Ngs1 (1-752 aa, including the GH3 domain and GNAT domain). The chimera failed to increase the acetylation level of H3K9 in *gcn5* mutant in glucose, but led to a significant increase in H3K9 acetylation in response to GlcNAc; although it is insufficient to restore the full HAT activity of Gcn5 (Fig. 4f). We further showed that mutating the predicted GlcNAc binding sites (K175H176) in Ngs1 blocked GlcNAc-induced acetylation on promoter chromatin H3K9 and H3K14 (Fig. 4d). Therefore, our data demonstrated that GlcNAc binding to the GH3 homology domain of Ngs1 is necessary for its HAT activity of GNAT domain, which is required for GlcNAc signalling.

### Conservation of Ngs1 in diverse fungi

**Figure 5:**
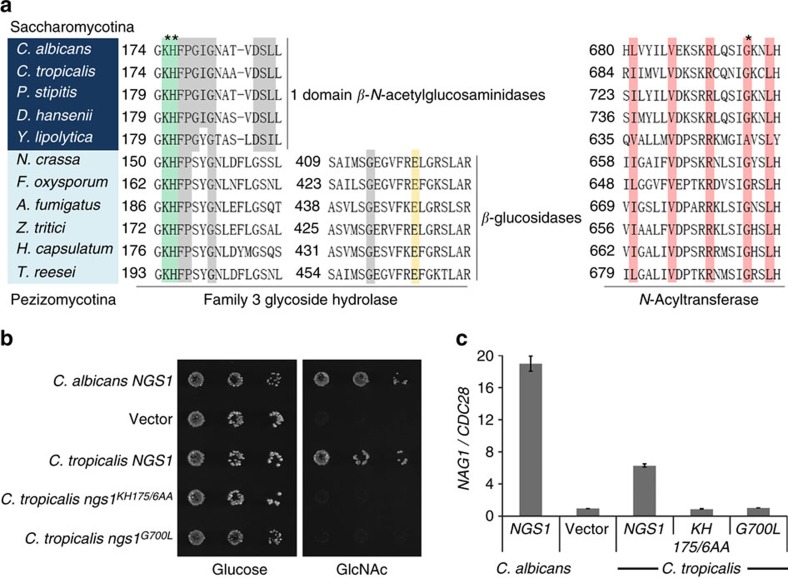
Evolutionary conservation of GlcNAc signalling in Ascomycota. (**a**) Partial multiple sequence alignment of Ngs1 orthologs in the GH3 glycosidase domain and the *N-*Acyltransferase domain from Saccharomycotina (shaded in dark blue) and Pezizomycotina (shaded in light blue). The conserved residues in one domain *β-N-*acetylglucosaminidases and two domain *β*-glucosidases, as determined by Litzinger and Fischer^29^, are shaded in grey. The residues known to interact with GlcNAc (shaded in green) and the glutamate residue known to act as the acid/base catalyst in *β*–glucosidases subfamily (shaded in yellow) are indicated. Residues shaded in pink in the *N-*Acyltransferase domain are conserved in motif A, as shown in Fig. 4a. The asterisks indicate the positions used in the mutational analysis in *C. tropicalis.* (**b**) Ectopically expressed *CtNGS1* suppresses the growth defect of *Cangs1* mutant on GlcNAc. Dilutions of *C. albicans ngs1* mutant cells carrying *CaNGS1* (HLY4395), *CtNGS1* (HLY4456), *Ctngs1*^*KH175/6AA*^ (HLY4457), *Ctngs1*^*G700L*^ (HLY4458) or vector alone were grown on YNB plates containing 2.5 mM GlcNAc or glucose at 30 °C for 2 days. (**c**) The conserved key GlcNAc binding sites K175H176 and acetyl-CoA interacting residue G700 in CtNgs1 are required for the induction of *NAG1* in *C. albicans*. *NAG1* mRNA levels of the strains from (**b**) were determined. Cells were grown in liquid SC medium supplemented with 2.5 mM GlcNAc at 30 °C and collected at 15 min for qRT-PCR analysis. Mean data±s.d. from three independent qRT-PCR experiments was plotted.

We performed BLAST searches to analyse the distribution of Ngs1 orthologs in fungi and found that Ngs1 orthologs are in many species belonging to the two subphyla, *Saccharomycotina* and *Pezizomycotina*, as recently reported^6^. Similar to the conservation of GlcNAc catabolic genes in the *Saccharomycotina* class, Ngs1 orthologs are found in organisms most closely related to *C. albicans* and missing in yeast that are more related to *S. cerevisiae*^3^,^46^. All the Ngs1 orthologs in the *Saccharomycotina* class carry an N-terminal domain with a high homology to the GH3 *β*-*N*-acetylglucosaminidase and a C-terminal region with a weak homology to the NAT domain in the GNAT family (Fig. 5a). Like *C. albicans* Ngs1, the conserved GlcNAc binding residues in the *β*-*N*-acetylglucosaminidase domain are present, whereas the Asp-His dyad residues required for the catalytic function and the binding site for the lactyl moiety of anhMurNAc are divergent in the Ngs1 orthologs. BLASTP analysis also identified Ngs1 orthologs throughout many different species of Pezizomycotina, such as Git7 in *Histoplasma capsulatum*^4^ and Nag3 in *T. reesei*^6^. Interestingly, Ngs1 orthologs are often present in the GlcNAc utilization gene cluster in filamentous fungi (Pezizomycotina)^6^, but Ngs1 is not associated with this cluster in *C. albicans*. Further, the transcriptional induction by GlcNAc was found for *H. capsulatum GIT7* and *T. reesei NAG3,* but not for *C. albicans NGS1* (Supplementary Fig. 2b). Ngs1 orthologs in Pezizomycotina contain the highly conserved GH3 domain at the N-terminus and NAT domain at the C-terminus. The GH3 domain carries conserved GlcNAc binding residues (Fig. 5a, shaded in green) and a glutamate acting as the acid/base catalyst in *β*–glucosidases subfamily (Fig. 5a, shaded in yellow)^47^, and therefore may retain the hydrolase activity. The Ngs1 orthologs in Pezizomycotina class are expected to have the ability to recognize GlcNAc monomer and transduce the GlcNAc signal, as deletion of *NAG3* in *T. reesei* resulted in a growth defect on GlcNAc (ref. 6).

To further determine if the function of Ngs1 orthologs is conserved in fungi, we examined the ability of *Candida tropicalis* Ngs1 to complement the GlcNAc utilization defect in *C. albicans ngs1* mutant. The cross-species experiment showed that ectopically expressed *CtNGS1* rescued the growth defect in the *Cangs1* mutant on GlcNAc (Fig. 5b), and partially rescued the defects in the induction of a GlcNAc catabolic gene (Fig. 5c). Furthermore, the conserved GlcNAc binding sites (K175H176) and acetyl-CoA interacting residue (G700) in CtNgs1 are necessary for its function in GlcNAc signalling (Fig. 5b,c). Altogether, the results suggest that the Ngs1-mediated GlcNAc sensing and signal transduction is conserved in fungi.

### Rep1 recruits Ngs1 to promoters

**Figure 6:**
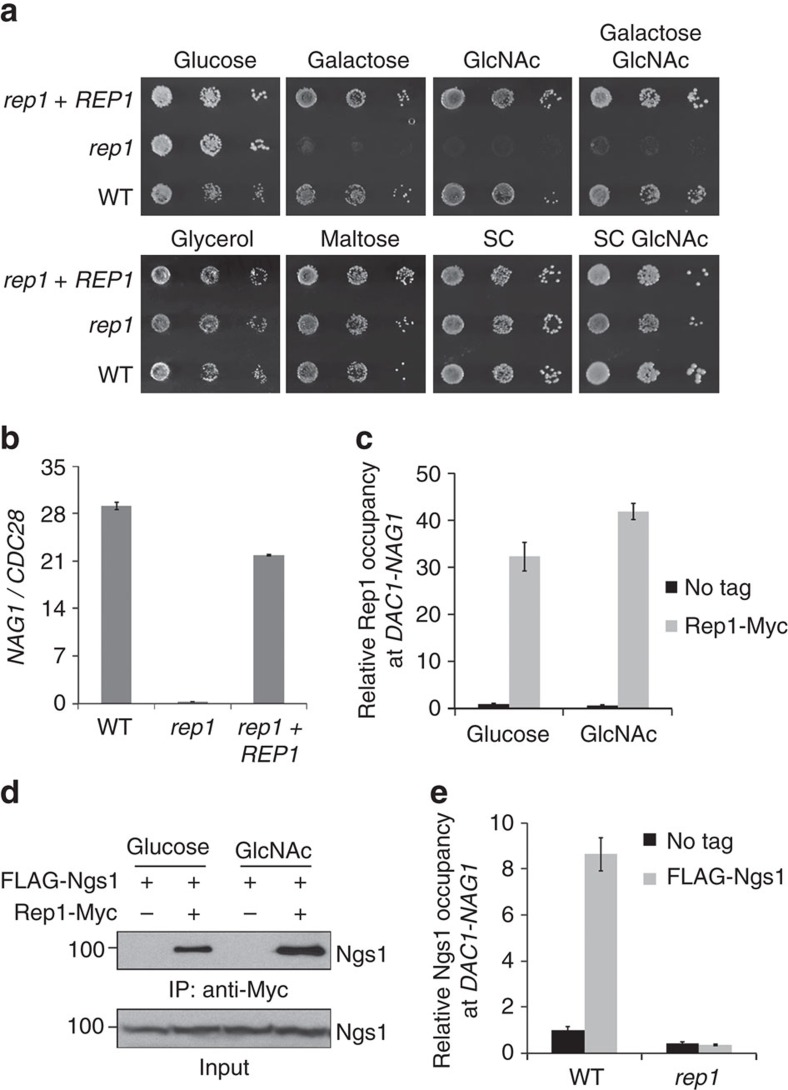
Rep1 recruits Ngs1 to the promoters of GlcNAc catabolic genes. (**a**) *REP1* is required for growth on GlcNAc and galactose in *C. albicans*. Dilutions of the *rep1Δ/rep1Δ* mutant (YLO141), *rep1Δ/rep1Δ::REP1* (YLO142), and the wild type (SC5314) cells were spotted onto YNB solid medium containing 2.5 mM of the indicated sugar or 5% glycerol. Cells were also tested for growth on SC solid medium in the absence or presence of 2.5 mM GlcNAc. Photographs were taken after 2 days of growth at 30 °C. (**b**) qRT-PCR analysis of *NAG1* transcription upon GlcNAc induction. Cells of the above three strains were grown in liquid SC medium with 2.5 mM GlcNAc for 2 h at 30 °C for RNA extraction. (**c**) ChIP of Rep1 at the promoter of *NAG1* and *DAC1*. The *rep1* mutant strain expressing Rep1-Myc or untagged Rep1 was grown in SC medium with GlcNAc or glucose at 30 °C, and cells were collected at 30 min for the ChIP experiment. The ratio of *NAG1-DAC1* promoter IP (bound/input) versus *ACT1* promoter IP (bound/input) in *rep1* mutant cells carrying untagged Rep1 in glucose medium was set to 1.00. (**d**) Ngs1 interacts with Rep1. Protein lysates from strains HLY4454 (FLAG-Ngs1, Rep1-Myc) and HLY4402 (FLAG-Ngs1) were subjected to immunoprecipitation with anti-Myc antibody, and the precipitated proteins were probed with anti-FLAG antibody. As an input control, cell lysates were analysed by Western blotting with the anti-FLAG antibody. (**e**) Ngs1 association at the *NAG1-DAC1* promoter depends on Rep1. For ChIP of Ngs1 in the wild-type strain (HLY4459) or the *rep1* mutant (HLY4453), cells were grown in SC GlcNAc medium at 30 °C for 30 min. The ratio of *NAG1-DAC1* promoter IP (bound/input) versus *ACT1* promoter IP (bound/input) in wild-type cells carrying untagged Ngs1 was set to 1.00. All data showed the average of three independent qPCR data with error bars representing the s.d.

The GlcNAc utilization gene cluster in filamentous fungi (*Pezizomycotina*) frequently harbours a transcription factor with an Ndt80-like DNA-binding domain, which is required for GlcNAc-induced expression of catabolic genes in *T. reesei*, designated as Ron1 (ref. 6). Its ortholog in *C. albicans* is Rep1, a negative regulator of *MDR1* transcription^48^. We found that, similar to Ron1 in *T. reesei,* Rep1 was essential for growth on GlcNAc (Fig. 6a), and transcriptional induction of GlcNAc catabolic genes (Fig. 6b). The defect of the *rep1* mutant in GlcNAc signalling is not due to a lack of *NGS1* expression (Supplementary Fig. 6a). Rep1 is tagged with myc at the C-terminus and is functional (Supplementary Fig. 1b). ChIP of Rep1-myc showed that Rep1 was present at the promoter region of *NAG1* and *DAC1* in a GlcNAc-independent manner, like Ngs1 (Fig. 6c). Immunoprecipitation of Rep1-myc showed that Rep1 could interact with Ngs1 in cells grown in either glucose or GlcNAc medium (Fig. 6d). Furthermore, the promoter recruitment of Ngs1 was dramatically reduced in the *rep1* mutant (Fig. 6e), and GlcNAc-inducible H3K9 and H3K14 acetylation at the promoter chromatin was abolished in the *rep1* mutant (Supplementary Fig. 6b). These results suggest that the Rep1 transcription factor recruits Ngs1 to the promoters of GlcNAc catabolic genes. Different from the *ngs1* mutant, the *rep1* mutant was also unable to grow on galactose (Fig. 6a). This suggests that Rep1 is not only the transcription factor for GlcNAc signalling, but also for galactose signalling.

### Ngs1 also functions in other GlcNAc-induced transcription

**Figure 7:**
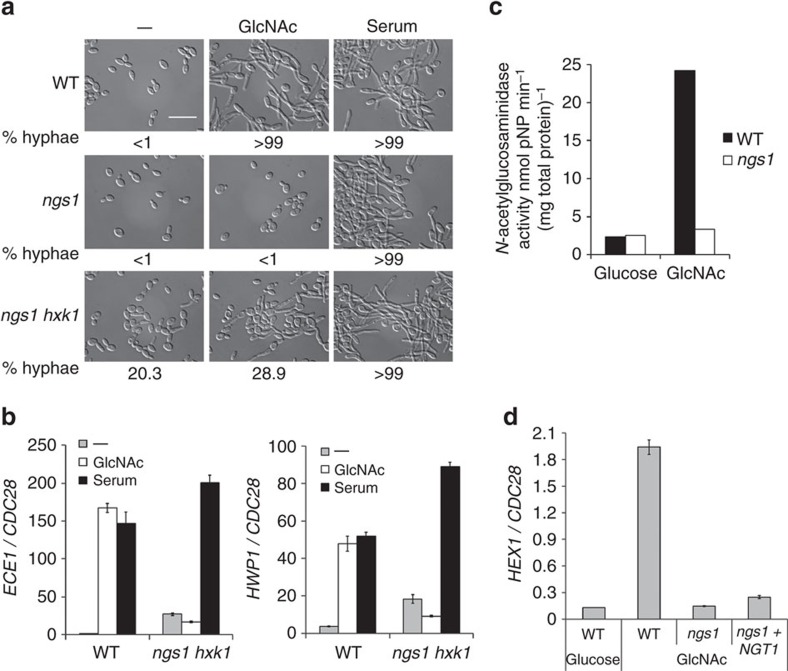
Ngs1 is essential for GlcNAc-induced hyphal transcription and *HEX1* expression. Morphology (**a**) and qRT-PCR analysis (**b**) of indicated strains during hyphal induction. Cells of wild type (SC5314), *ngs1* (HLY4391) and *ngs1 hxk1* double mutant (HLY4427) were grown to log phase at 30 °C, and then treated with either 50 mM GlcNAc or 10% serum. Cells were collected after incubation for 3.5 h at 37 °C. The percentage of hyphal cells was determined by counting at least 200 cells per sample. The average length of hyphae was measured to show the difference between wild type and *ngs1 hxk1* double mutant in response to GlcNAc. Wild-type cells formed long hyphae after 3.5 h growth in GlcNAc-containing medium (46.4±12.9 μm; mean±s.d.). Hyphae length of the *ngs1 hxk1* double mutant was shorter, 15.6±5.1 μm (mean±s.d.) in the medium without GlcNAc and 23.7±9.3 μm (mean±s.d.) upon GlcNAc induction. For all images: Scale bar, 20 μm. Expression level of *ECE1* and *HWP1* was quantified by qRT-PCR and normalized with *CDC28*. (**c**) The effect of *NGS1* on GlcNAc-induced *N*-acetylglucosaminidase activity in *C. albicans* cells. Wild type (SC5314) and *ngs1* mutant (HLY4391) cells were resuspended in YEP medium containing 50 mM GlcNAc or glucose at OD_600_=0.3, and *N*-acetylglucosaminidase activity was measured after incubation at 30 °C for 2 h. Enzyme activities are the means of three determinations that did not vary more than 15%. (**d**) Expression analysis of *HEX1*. Cells of wild type (SC5314), *ngs1* mutant (HLY4396), as well as *ngs1* mutant carrying *NGT1* under the *ADH1* promoter (HLY4393) were incubated in liquid SC medium with 2.5 mM GlcNAc or glucose for 2 h at 30 °C. *HEX1* expression levels were quantified by qRT-PCR and normalized with *CDC28*. All data showed the average of three independent qRT-PCR experiments with error bars representing the s.d.

Having determined the essential role of Ngs1 in GlcNAc sensing and transcriptional activation of GlcNAc catabolic genes, we then investigated whether Ngs1 plays a role in GlcNAc-induced hyphal development. The *ngs1 hxk1* double mutant was constructed and used to examine the function of Ngs1 in hyphal growth because GlcNAc is inhibitory to the growth of the *ngs1* mutant at 37 °C (Supplementary Fig. 7). The *hxk1* mutant can grow and develop hyphae as wild type in GlcNAc containing media where GlcNAc is not the only carbon source^26^. The fact that the inhibitory effect could be removed by *HXK1* deletion suggested that the reported increase in *HXK1* expression at 37 °C (ref. 49) might lead to the accumulation of GlcNAc-6-PO_4_ in the *ngs1* mutant, resulting in growth inhibition as reported for the *dac1* or *nag1* mutants^26^ (Supplementary Fig. 7). Hyphal growth was induced by directly adding GlcNAc or serum into log-phase cultures to avoid inoculation-induced hyphal initiation^50^. The wild-type strain developed extensive filaments after 3.5 h of hyphal induction by serum or GlcNAc. *ngs1* single mutant and *ngs1 hxk1* double mutant cells also developed hyphae in serum similar to the wild-type strain (Fig. 7a). However, the *ngs1 hxk1* double mutant displayed defective hyphal development in response to GlcNAc with a mixture of short heterogeneous filaments and over 70% yeast cells (Fig. 7a). Furthermore, although a slightly higher level of hyphal formation was observed in the *ngs1 hxk1* double mutant upon GlcNAc induction, the expression of hypha-specific genes, such as *ECE1* and *HWP1*, were not induced in the double mutant (Fig. 7b), which may reflect the transcription-independent hyphal morphogenesis reported by Naseem *et al*.^17^. The *ngs1 hxk1* double mutant in yeast condition displayed yeast form with a low percentage (about 20%) of germ tube, a phenotype in agreement with a previous report that deletion of *HXK1* caused a high tendency to form hyphae^49^. Therefore, Ngs1 is important for GlcNAc-induced hyphal development, especially hyphal transcription.

Recently, it has been shown that *C. albicans* can obtain GlcNAc during organ colonization by secreting Hex1 as a carbon scavenger^14^. GlcNAc induces the expression of *HEX1*, which encodes a secreted *N*-acetylglucosaminidase^51^,^52^. We detected *N*-acetylglucosaminidase activity in cells exposed to GlcNAc, and the activity was abolished by deletion of *NGS1* (Fig. 7c). Since *N*-acetylglucosaminidase activity in *C. albicans* was shown to be Hex1-dependent and GlcNAc inducible^51^, we reasoned that the Ngs1-dependent and GlcNAc inducible *N*-acetylglucosaminidase activity we detected in *C. albicans* was due to Ngs1-mediated transcriptional induction of *HEX1* expression. RT-PCR analysis showed that no significant induction of *HEX1* was observed in the *ngs1* mutant in GlcNAc medium (Fig. 7d). Further, overexpression of *NGT1* was unable to promote *HEX1* expression in the *ngs1* mutant in GlcNAc. Thus, Ngs1-mediated intracellular GlcNAc signal transduction also controls *HEX1* expression.

## Discussion

**Figure 8:**
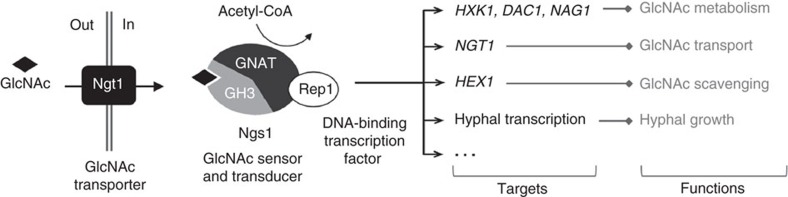
Model of GlcNAc signalling in *C. albicans.* GlcNAc enters the cell via the GlcNAc-specific transporter Ngt1 (ref. 3). Ngs1 acts as the sensor and signal transducer via its N-terminal GlcNAc binding domain and C-terminal GNAT domain, as well as the transcription factor Rep1 for promoter targeting. Binding of GlcNAc to Ngs1 activates the *N*-acetyltransferase activity, resulting in promoter histone acetylation and transcription of all GlcNAc-induced genes.

GlcNAc is ubiquitously present as a component of the cell surface on a wide range of cells from bacteria to humans. To utilize the GlcNAc in the biosphere, microbes have developed various strategies for GlcNAc scavenging and utilization. Some microbes have even evolved to link GlcNAc signalling with cell fate development and virulence properties. Central to GlcNAc metabolism is how microbes sense GlcNAc in the environment and respond with a proper transcriptional program. For bacteria, extracellular GlcNAc is taken into cells using a phosphotransferase system that converts GlcNAc to GlcNAc-6-PO_4_ (ref. 1). Intracellular GlcNAc-6-PO_4_ regulates the expression of GlcNAc-induced genes by causing allosteric changes of a global transcriptional regulator in its binding affinity for the target genes^2^,^53^,^54^. Fungi use the GlcNAc transporter Ngt1 of the major facilitator superfamily transporters for GlcNAc uptake^3^,^4^,^6^. Here we show that *C. albicans* uses Ngs1 as the master regulator of GlcNAc signalling that can sense intracellular GlcNAc via its N-terminal GlcNAc binding domain. The GlcNAc-binding is necessary for chromatin histone acetylation through its C-terminal GNAT domain. Ngs1 controls not only the induction of GlcNAc catabolic genes; it also controls GlcNAc-induced expression of *NGT1*, *HEX1* and hyphal genes, which are for GlcNAc uptake, scavenging and pathogenesis, respectively (Fig. 8). Targeting of Ngs1 to specific promoters is mediated through the transcriptional factor Rep1. Both Ngs1 and Rep1 are conserved in fungi, and their homologues are required for GlcNAc signalling and catabolism in the filamentous fungus *T. reesei*^6^. The *C. tropicalis* Ngs1 has the same function and regulation. Therefore, the GlcNAc signalling pathway in fungi consists of the transporter Ngt1, the sensor-transducer Ngs1, and the transcription factor Rep1/Ron1 (Fig. 8). The evolution of GlcNAc regulation of promoter histone acetylation in fungi, in comparison to direct regulation of transcription factor affinity on promoter DNA in bacteria, probably reflects the need to overcome the repressive barrier imposed by the chromatin in eukaryotes.

There is growing evidence that the molecular scaffolds employed by enzymes of sugar metabolism are ideally suited to function as ligand sensors in transcriptional regulation. For example, the Gal3 transducer of the GAL regulon in *S. cerevisiae* has ∼90% sequence similarity to galactokinase Gal1, but does not possess galactokinase activity. Gal3 interacts with the Gal80 repressor only when Gal3 is in a ‘closed' state induced by galactose binding^55^. Another example is the negative transcriptional regulator NmrA in *Aspergillus nidulans.* The structure of NmrA shows unexpected similarity to a superfamily of short-chain dehydrogenase/reductases^56^. Similarly, NmrA has lost the conserved catalytic residue for enzyme activity, but appears to have adapted to use the scaffold for transcriptional regulation. The N-terminal domain of Ngs1 is very similar in structure to bacterial NagZs of the single-domain GH3 *β*-*N*-acetylglucosaminidases^34^. The binding coordinates of NagZs for GlcNAc are conserved in Ngs1 (ref. 36), and the interaction of Ngs1 with GlcNAc is essential for GlcNAc signalling in *C. albicans*. The GlcNAc binding domain is conserved in Ngs1 orthologs in fungi. Like Gal3 and NmrA, Ngs1 lacks hydrolase activity, and the conserved catalytic residues in NagZs are lost in Ngs1 and not required for GlcNAc signalling in *C. albicans*. We propose that Ngs1 does not function as a hydrolase in *C. albicans*. Rather, the conserved GlcNAc binding domain in Ngs1 functions as a GlcNAc sensor that modulates the activity of Ngs1 upon GlcNAc binding.

Ngs1 is a new member of the GCN5-related *N*-acetyltransferase (GNAT) family. The conserved sites for Acetyl-CoA binding in the GNAT domain of Ngs1 are required for GlcNAc signalling and H3K9 and H3K14 acetylation at the promoter of GlcNAc catabolic genes. Ngs1 is a unique GNAT because its activity is induced by GlcNAc. In *C. albicans*, Gcn5 is responsible for almost all of acetylation at H3K9, but it is not required for GlcNAc-induced H3K9 acetylation at the promoters of GlcNAc catabolic genes. GlcNAc-induced H3K9ac is only detected at specific promoters, but not detectable when assayed with total histone, even in *gcn5* mutant cells where the basal H3K9ac is low. This is likely due to a lack of a structural determinant necessary for acceptor substrate specificity in Ngs1. In support of this, the GNAT domain of Ngs1 is capable of increasing the total level of H3K9ac in response to GlcNAc when its C-terminus is replaced with the bromodomain and an Ada2 interaction domain of Gcn5. These domains of Gcn5 may allow the integration of the chimeric protein into the SAGA and ADA HAT complexes to acetylate nucleosomal histones. Recruitment of Ngs1 by Rep1 to the promoters of GlcNAc-inducible genes targets Ngs1 to specific locations for chromatin histone acetylation. Therefore, we suggest that GlcNAc-induced chromatin H3 acetylation by Ngs1 is limited to specific chromatin locations bound with Rep1. As *ngs1* and *rep1* mutants showed same defects, the major function of Ngs1 in GlcNAc signalling should be chromatin histone acetylation and transcriptional regulation, although we cannot exclude the possibility of Ngs1 acetylating other proteins or small molecules. Like Gcn5 (ref. 10) and NatA (ref. 57), complex formation may be necessary for Ngs1 in substrate selection. Interestingly, the domain layout in Ngs1 is similar to the mammalian O-GlcNAcase (OGA), which is responsible for removal of O-linked GlcNAc from nucleocytoplasmic proteins. Like Ngs1, OGA has a glycoside hydrolase (GH) domain at the N-terminus and a GNAT-like fold at the C-terminus^58^. However, the OGA activity resides in the GH domain and its GNAT domain has lost some conserved sites for Acetyl-CoA binding. Therefore, Ngs1 is conserved in fungi, but not in multicellular organisms. It is the first GNAT found to respond directly to the availability of a sugar in the environment and control cellular transcriptional programs.

Rep1 is required for GlcNAc signalling and gene expression, as reported for Ron1 in the filamentous fungus *T. reesei*^6^. We demonstrate that Rep1 is the DNA-binding transcription factor that recruits Ngs1 to specific promoters for GlcNAc-induced histone acetylation and transcription. Interestingly, Rep1 is also required for galactose utilization, and GlcNAc can induce the expression of galactose metabolic genes in *C. albicans*^26^. The galactose signalling pathway is rewired in *C. albicans* and is not regulated by the Gal3, Gal80 and Gal4 regulatory circuitry^59^. In fact, the growth defect of the *rep1* mutant is stronger than known mutants involved in galactose signal transduction^59^,^60^. Unlike the *rep1* mutant, the *ngs1* mutant can grow on galactose. This indicates that Rep1 can recruit other regulators for galactose signalling in *C. albicans.*

Ngs1 represents the first example of how one protein can sense the availability of a sugar taken from outside the cell and directly regulate transcription by modulating its GNAT activity. The GlcNAc sensor-transducer is a master regulator of GlcNAc signalling in fungi that controls GlcNAc catabolism, morphogenesis and pathogenesis.

## Methods

### Media and growth conditions

*C. albicans* strains were routinely grown at 30 °C in YPD (2% Bacto peptone, 2% glucose, 1% yeast extract). Transformants were selected on synthetic medium (0.17% Difco yeast nitrogen base w/o ammonium sulphate, 0.5% ammonium sulphate and auxotrophic supplements) with 2% glucose. The ability of cells to grow was tested by spotting dilutions of cells onto YNB (0.17% Difco yeast nitrogen base w/o ammonium sulphate, 0.5% ammonium sulphate) or SC (0.17% Difco yeast nitrogen base w/o ammonium sulphate, 0.5% ammonium sulphate, complete supplement mixture of amino acids) solid media with 2.5 mM of different sugars followed by incubation at 30 °C for 2 days. To determine the expression of GlcNAc catabolic genes, cells were grown overnight in liquid YPD at 30 °C, pelleted, washed three times in PBS, diluted 1:50 in SC medium with different sugars, and incubated for 2 h at 30 °C.

Hyphal development is induced in cells growing in log phase without inoculation to block the effect of farnesol depletion on hyphal induction as described previously^26^. *C. albicans* cells grown overnight at 30 °C in YPD were washed three times with PBS, resuspended in an equal volume of PBS, and diluted 1:100 into liquid SC medium containing 50 mM galactose. After 4 h of incubation at 30 °C, hyphal growth was induced by a shift in temperature to 37 °C in combination with 50 mM GlcNAc. In addition to GlcNAc, 10% Serum was used for morphology assay in this study as well.

### Screening for mutants defective in growth on GlcNAc

The deletion mutant library affecting 674 genes of *C. albicans*^24^ and the wild type reference strain SN250 were grown overnight in liquid YPD at 30 °C. Then cells were diluted 1:200 fold into sterile PBS in 96-well plates. The ability of cells to grow on different sugars was tested by spotting diluted cells onto YNB solid media with 2.5 mM glucose or 2.5 mM GlcNAc followed by incubation at 30 °C. The medium was supplemented with 0.1 mg ml^−1^ arginine to permit the growth of mutant strains in library. Three mutants could not grow on the GlcNAc-containing medium. However, these three mutants were able to grow to the same extent as the wild-type strain on glucose after incubation for 2 days.

### Plasmid and strain construction

SC5314 genomic DNA was used as the template for all PCR amplifications of *C. albicans* genes. The *C. albicans* strains used in the present study are listed in Supplementary Table 2. The primers used for PCR amplification are listed in Supplementary Table 1. *C. albicans ARG4* was amplified with primers as previously described^61^, and then transformed into the *ngs1* mutant strain^24^. Transformants were selected on arginine dropout medium. Arg^+^ transformants (HLY4391) were streaked onto 5-fluoroorotic acid plates to select for the Ura^–^ strain (HLY4392).

pBES116-NGS1 was constructed by inserting a 3.78-kb PCR fragment (primers 1 and 2) containing the *NGS1* promoter and coding sequence into the NotI-PstI site of pBES116 (ref. 62). AscI-digested pBES116-NGS1 was introduced into *ngs1* mutant (HLY4392) to express *NGS1* under the endogenous promoter. Two-step PCR was used to create pBES116-NGS1^D74A^. Two pairs of primers (primers 1 and 3, 2 and 4) were used to PCR amplify overlapping *NGS1* fragments with the mutation in the overlapping region. The PCR products were purified and mixed as templates for another round of PCR amplification using the primers 1 and 2, which produced the full-length *NGS1*^*D74A*^ sequence. The resulting mutant, *NGS1*^*D74A*^, was inserted into the NotI-PstI site of pBES116 to generate pBES116-NGS1^D74A^. The plasmid was digested with AscI for integration into the *ADE2* locus. This two-step PCR procedure was used to introduce mutations into *NGS1* in a pBES116-based construct. Mutations were confirmed by sequencing. Plasmids for expression of 3 × FLAG tag fusion proteins were constructed as follows. The promoter region of *NGS1* was amplified with primers 1 and 23 and inserted into the NotI-PstI site of pBES116 to generate pBES116-NGS1p. PCR products (primers 24 and 2) containing the entire *NGS1* coding region and a 3 × FLAG tag were amplified from constructs pBES116-NGS1 and pBES116-NGS1 with mutations. The DNA fragments were then inserted into pBES116-NGS1p at the PstI site. The resulting plasmids were digested with AscI and introduced into *ngs1* mutant strain (HLY4392) to express the 3 × FLAG fusion proteins under the endogenous promoter. A 2.9-kb PCR product (primers 27 and 28) containing the entire *NGS1* coding region was inserted into the MluI site of Mal2-GFP-HGC1 (ref. 61) to create MAL2p-GFP-NGS1. The AscI-digested MAL2p-GFP-NGS1 DNA fragment was introduced into the *ngs1* mutant strain (HLY4392) for GFP-Ngs1 expression. A 1.91-kb PCR product (primers 1 and 31) containing the *NGS1* promoter and N-terminal *NGS1* coding region was inserted into the NotI-PstI site of pBES116 to generate pBES116-NGS1N. The 3 × FLAG tagged N-terminal *NGS1* was amplified with primers 24 and 31 and inserted into pBES116-NGS1p at the PstI site. The C-terminal *NGS1* coding region was amplified using primers 32 and 33. The resulting PCR product was digested with PstI and KpnI and inserted into the PstI-KpnI site of pBES116-NGS1p to create pBES116-NGS1C. To express 3 × FLAG fusion protein, the C-terminal *NGS1* was amplified using primers 44 and 33 and then ligated with pBES116-NGS1p. The *NGS1* ortholog in *Candida tropicalis* (CTRG_01063) was obtained by PCR (primers 38 ad 39) using genomic DNA from ATCC 750. The resulting PCR product was digested with PstI and EcoRV and inserted into the pBES116-NGS1p to create pBES116-CtNGS1. Two-step PCR was used to produce pBES116-CtNGS1^KH175/176AA^ and pBES116-CtNGS1^G700L^.

To create a chimera comprising the Ngs1 GNAT domain fused to the ADA2 interaction domain and bromodomain in Gcn5, we used a strategy employing PCR-mediated recombination so that no restriction sites at the junction of the chimera are needed. Primers 46 and 47 were designed in a way that both PCR products shared the same sequence at one end. The PCR product (primers 45 and 48) corresponding to Ngs1 aa 595 to 752 plus Gcn5 aa 253 to 450 was inserted into the BamHI-MluI site of pPR671-SAT1 (ref. 63) to create pPR671-chimera-SAT1. The SacI-digested pPR671-chimera-SAT1 fragment was integrated into the *NGS1* locus in the *gcn5* mutant. The *REP1* coding sequence (primers 49 and 50) was inserted into the BamHI-MluI site of pPR671-SAT1 and p673 (ref. 64) to generate plasmids pPR671-REP1-SAT1 and pPR673-REP1. The resulting constructs were digested with StuI to integrate into the genomic *RP10* locus for expression of 13 × Myc fusion proteins under the *ACT1* promoter.

*NGT1* was deleted using *SAT1*-flipping strategy^65^. Upstream (primers 13 and 14) and downstream (primers 15 and 16) sequences of *NGT1* were cloned as ApaI-XhoI and NotI-SacII fragments, respectively, on both sides of the *SAT1* flipper cassette to obtain the plasmid pSFS2-NGT1 for *NGT1* disruption. To disrupt the endogenous copies of *NGT1* in wild-type cells, pSFS2-NGT1 was linearized using ApaI and SacII, which was followed by two sequential rounds of transformation, selection, and recycling of the *SAT1* marker. pBA1-NGT1 was constructed for expression of *NGT1* under the control of *ADH1p* in *C. albicans*. The full-length coding sequence of *NGT1* was amplified with primers 25 and 26 and inserted into the ClaI-KpnI site of pBA1 (ref. 66) to generate pBA1-NGT1.

*HXK1* deletion in *ngs1* mutant strain was based on PCR recombination by the method of Wilson *et al*.^67^. Primers 34 and 35 were used to amplify *C*. *albicans URA3* and *ARG4* from plasmids pGEM-URA3 and pRS-ARG4ΔSpeI, respectively. The *ngs1* mutant strain^24^ was streaked onto 5-fluoroorotic acid plates to select for the Ura^–^ strain. The first copy of *HXK1* was disrupted by the transformation of *ARG4* into the *ngs1* mutant (Ura^–^) strain. *C. albicans URA3* was used to replace the second copy of *HXK1*. All deletions were confirmed by PCR.

### Microscopy

Cells of *ngs1* mutant carrying GFP-Ngs1 under the *MAL2* promoter and wild-type strain were grown overnight to log phase in liquid SC medium containing 50 mM maltose, and then treated with or without 50 mM GlcNAc for 30 min. GFP-Ngs1 was detected by fluorescence microscopy, and cell morphology was detected using differential interference contrast (DIC) optics. Images were obtained on an inverted Zeiss Axio Observer.Z1 microscope (Carl Zeiss MicroImaging, Inc., Thornwood, NY) fluorescent system equipped with an X-Cite series 120 mercury lamps. DAPI (4′,6-diamidino-2-phenylindole) stain was applied for the visualization of the cell nucleus. Each photomicrograph represents the majority of the cells.

### Quantitative PCR expression analysis

Total RNA was purified from *C. albicans* cells using the RNeasy Mini kit and DNase-treated at room temperature for 15 min using the RNase-free DNase Set (Qiagen), cDNA was synthesized using the SuperScript II Reverse Transcriptase kit (Invitrogen), and qPCR was done using the iQ SYBR Green Supermix (Bio-Rad). The primers for *HXK1*, *DAC1, NGT1* and *NAG1* were used as previously described^26^. Primers for *HWP1* and *ECE1* were used as described by Lu *et al*.^64^. Primers for *HEX1* were used as described by Ruhela *et al*.^14^. Primers 51 and 52 were used to amplify *C*. *albicans NGS1.* Primers 29 and 30 were used to amplify *C*. *albicans CDC28.* The signals obtained from *CDC28* mRNA were used for normalization. All data showed the average of three independent qRT-PCR experiments with error bars representing the s.d.

### Chromatin immunoprecipitation

Cells were grown overnight in liquid YPD at 30 °C, pelleted, washed three times in PBS, diluted 1:20 in SC medium supplemented with 10 mM GlcNAc or glucose, and collected at 30 min for Chromatin immunoprecipitation. ChIP experiments were performed as described with modifications^63^. DNA was sheared by sonication eight times for 20 s at high power on a Bioruptor (diagenode) with 40 s intervals. For the IP, 5 μl of anti-FLAG (F3165, Sigma), 10 μl of anti-Myc (SC-789, Santa Cruz), 4 μl of anti-H3 (ab1791, Abcam), 5 μl of anti-acetylated-H3K9 (06-942; Millipore) or 5 μl of anti-acetylated-H3K14 (ab52946; Abcam) antibodies were used for ∼2 mg of chromatin proteins in an immunoprecipitation volume of 300 μl. ChIP DNA was quantitated by qPCR with primers 17 and 18 at the promoter for *DAC1* and *NAG1*. The *ACT1* promoter (primers 36 and 37) served as a negative control. FLAG-Ngs1 and Rep1-Myc expressing strains were used with untagged strains as controls. The FLAG-Ngs1 or Rep1-Myc enrichment is presented as a ratio of *NAG1-DAC1* promoter IP (bound/input) versus control locus IP (bound/input). H3 ChIP signal was used to normalize H3K9ac and H3K14ac values. Levels of H3K9ac and H3K14ac at the *NAG1-DAC1* promoter were further normalized to the respective value at the *ACT1* promoter region. The ChIP data showed the average of three independent qPCR data with error bars representing the s.d.

### *N*-acetylglucosaminidase assay

*N*-acetylglucosaminidase activity was measured in whole cell extracts, using pNP-β-D-GlcNAc (4-Nitrophenyl N-acetyl-β-D-glucosaminide, Sigma) as a substrate. Overnight cultures of wild-type and *ngs1* mutant cells were washed three times in sterile PBS, resuspended in YEP (2% Bacto peptone, 1% yeast extract) medium containing 50 mM GlcNAc or glucose at OD_600_=0.3, and incubated at 30 °C for 2 h. Cells of each sample were pelleted at 3,000 g for 5 min at 4 °C, and resuspended in 0.35 ml of lysis buffer (50 mM Tris–HCl, pH 7.5, 150 mM NaCl, 0.1% NP40) plus protease inhibitor cocktail (Roche). Total protein was extracted using a Fast-Prep system (FastPrep-24; MP Biomedicals). Protein concentration was determined by the Bradford method. Equal amounts of proteins were incubated at 37 °C for 30 min in 0.25 ml volumes containing the following: 1 mg ml^−1^ pNP-β-D-GlcNAc, 50 mM sodium phosphate buffer, pH 5.5 and 50 mM NaCl. Reactions were stopped by the addition of 0.5 ml of 4% (wt per vol) Na_2_CO_3_, and the absorbance was measured at 405 nm. An extinction coefficient of 18,300 (M × cm)^−1^ was used to calculate *p*-nitrophenol produced per minute. One unit of enzyme was defined as that which catalysed the formation of 1 μmol of *p*-nitrophenol per min. No hydrolysis of pNP-β-D-GlcNAc was detected in the controls, containing boiled extracts of *C. albicans* cells.

### GlcNAc-agarose affinity chromatography

A total of 400 μl of protein extract from cells expressing wild-type or mutant 3FLAG-Ngs1 or untagged Ngs1 was incubated with 50 μl GlcNAc-agarose beads (A2278; Sigma) overnight at 4 °C under rotation. The beads were washed three times with TBS buffer (20 mM Tris-HCl, pH 7.5, 150 mM NaCl) to remove unbound protein. The bound protein was eluted from the matrix with 50 μl of 4% SDS for 30 min at 23 °C. The samples were collected by low-speed centrifugation. The input and bound fractions were analysed on Western blots. Uncropped scans of western blots are presented in Supplementary Fig. 8.

### Data availability

The authors declare that all data supporting the findings of this study are available within the article and its Supplementary Information, or from the corresponding author on request.

## Additional information

**How to cite this article:** Su, C. *et al*. N-acetylglucosamine sensing by a GCN5-related *N*-acetyltransferase induces transcription via chromatin histone acetylation in fungi. *Nat. Commun.* **7,** 12916 doi: 10.1038/ncomms12916 (2016).

## Supplementary Material

Supplementary Information Supplementary Figures 1-8, Supplementary Tables 1-2 and Supplementary References.
