# Supplementary material for: N-acetylglucosamine sensing by a GCN5-related N-acetyltransferase induces transcription via chromatin histone acetylation in fungi
Source: Nat Commun. 2016 Oct 3;7:12916. doi: 10.1038/ncomms12916 (PMC5063960; doi:10.1038/ncomms12916)
Supplement: Supplementary Information — Supplementary Figures 1-8, Supplementary Tables 1-2 and Supplementary References. [file ncomms12916-s1.pdf]

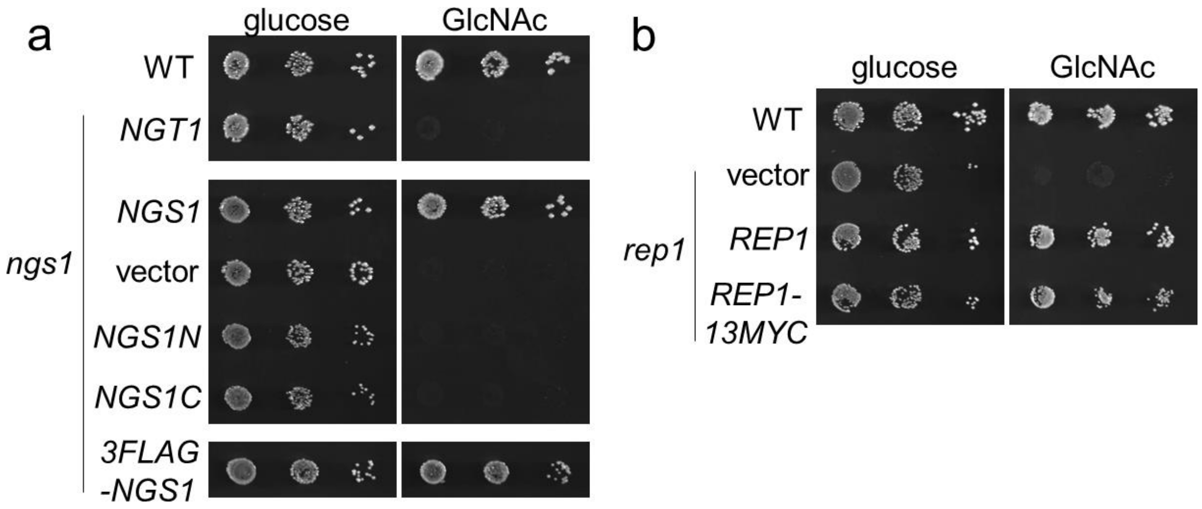

Supplementary Figure 1. Growth assay. (**a**) Cells of wild type (SC5314) and *ngs1* mutant carrying *ADH1p-NGT1*, *NGS1p-NGS1*, *NGS1p-NGS1N*, *NGS1p-NGS1C*, *NGS1p-3FLAG-NGS1,* or vector alone were serially diluted 10-fold, spotted onto YNB solid medium containing 2.5 mM GlcNAc or glucose, and incubated at 30°C for 2 d. (**b**) Cells of wild type (SC5314) and *rep1* mutant carrying wild-type *REP1*, C-terminally Myc-tagged *REP1*, or vector alone were serially diluted 10-fold, spotted onto YNB solid medium containing 2.5 mM GlcNAc or glucose, and incubated at 30°C for 2 d.

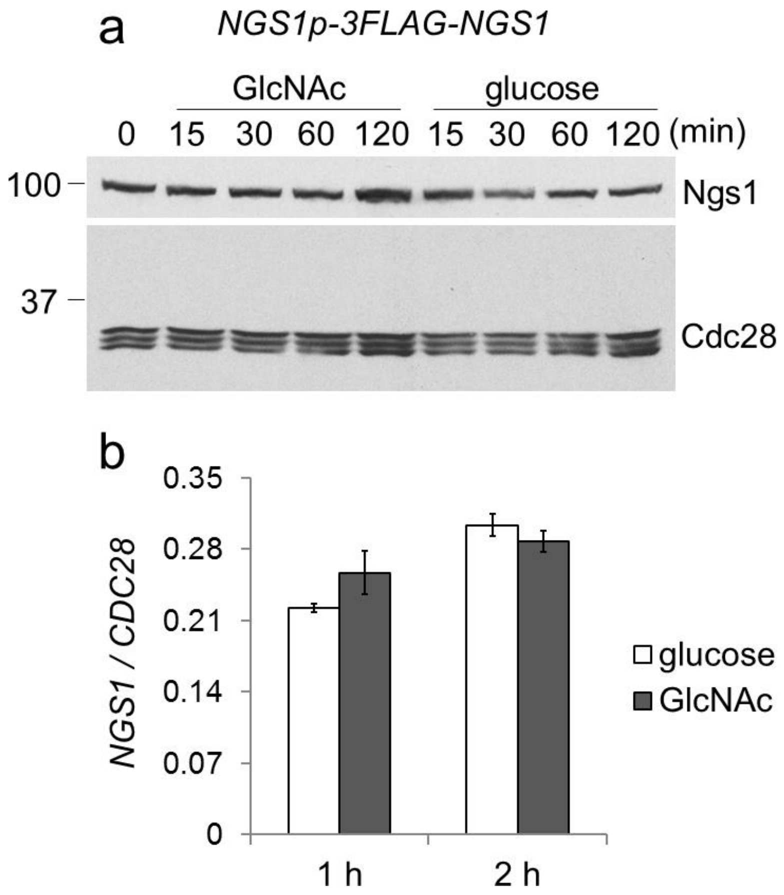

Supplementary Figure 2. The expression of *NGS1* upon GlcNAc induction. (**a**) A time-course analysis of Ngs1 expression by Western Blot. An overnight culture of *ngs1* mutant cells carrying 3FLAG-Ngs1 (HLY4402) was pelleted, washed three times in PBS, and diluted 1:50 fold into SC medium containing 20 mM GlcNAc or glucose at 30°C. Cells were collected at 0 min, 15 min, 30 min, 1 h, and 2 h for Western analysis. (**b**) RT-PCR analysis for *NGS1* expression. An overnight culture of wild-type cells was pelleted, washed three times in PBS, and diluted 1:50 in SC medium with 20 mM GlcNAc or glucose at 30°C. Cells were collected at 1 h and 2 h for RT-PCR analysis of *NGS1* expression. The signals obtained from *CDC28* mRNA were used for normalization. All data showed the average of three independent qRT-PCR experiments with error bars representing the SD.

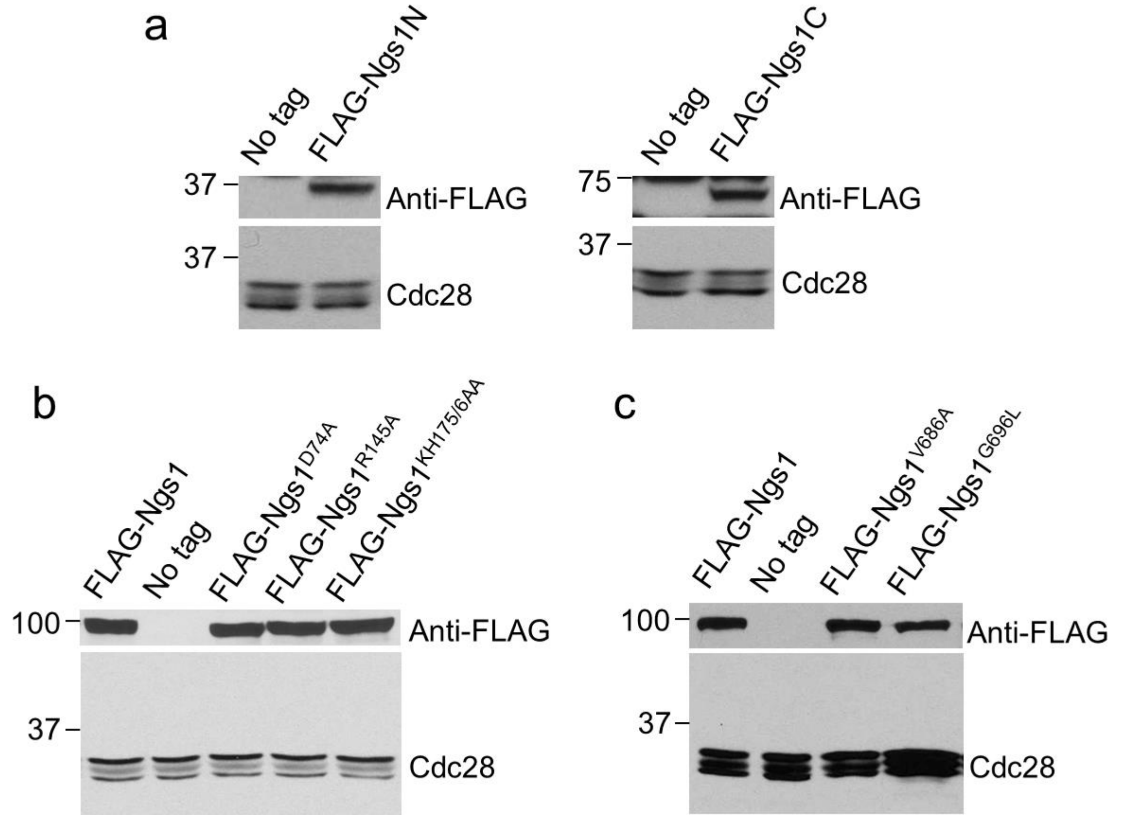

Supplementary Figure 3. Western analysis for truncated and mutant Ngs1. (**a**) Levels of 3FLAG-tagged proteins in *ngs1* mutant cells carrying 3FLAG-Ngs1N (HLY4460), 3FLAG-Ngs1C (HLY4461), or untagged Ngs1 (HLY4395) were analyzed via western blotting. Cells were incubated in SC medium containing 2.5 mM GlcNAc at 30°C. (**b**) *ngs1* mutant cells carrying 3FLAG-Ngs1 (HLY4402), 3FLAG-Ngs1^{D74A} (HLY4403), 3FLAG-Ngs1^{R145A} (HLY4404), 3FLAG-Ngs1$^{KH175/6AA}$ (HLY4405), or untagged Ngs1 (HLY4395) were incubated in SC medium containing 2.5 mM GlcNAc at 30°C. Levels of 3FLAG-tagged proteins were analyzed via western blotting. (**c**) Levels of 3FLAG-tagged proteins in *ngs1* mutant cells carrying 3FLAG-Ngs1 (HLY4402), 3FLAG-Ngs1^{V686A} (HLY4462), 3FLAG-Ngs1^{G696L} (HLY4463), or untagged Ngs1 (HLY4395) were analyzed via western blotting. Cells were incubated in SC medium containing 2.5 mM GlcNAc at 30°C.

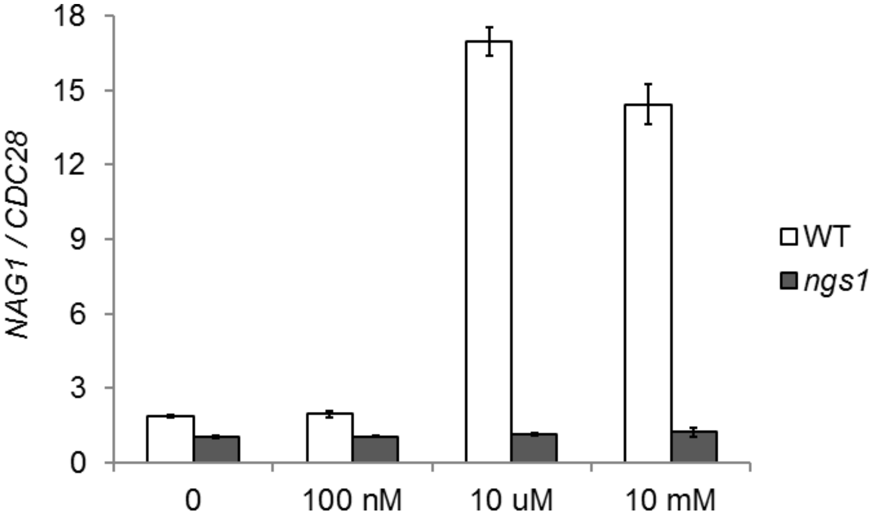

Supplementary Figure 4. Micromolar concentrations of GlcNAc were sufficient to promote *NAG1* expression. Overnight cultures of wild-type (SC5314) and *ngs1* mutant cells in liquid YPD were pelleted, washed three times in PBS, and inoculated at a 1:20 ratio into SC medium supplemented with increasing concentrations of GlcNAc at 30°C. Cells were collected at 15 min for qRT-PCR analysis of *NAG1* expression. The signals obtained from *CDC28* mRNA were used for normalization. All data showed the average of three independent qRT-PCR experiments with error bars representing the SD.

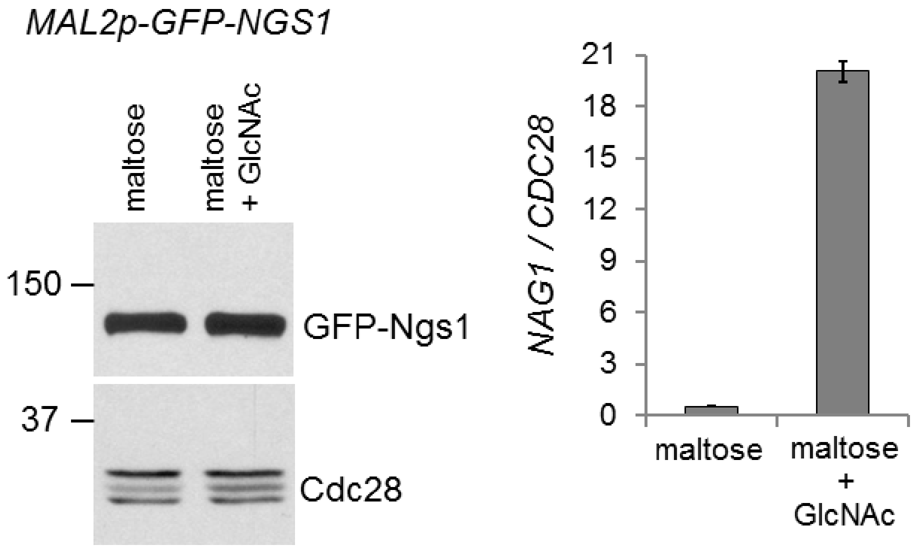

Supplementary Figure 5. The GFP-Ngs1 fusion is functional. The *ngs1* mutant strain expressing GFP-Ngs1 under the *MAL2* promoter (HLY4397) was grown to log phase in liquid SC medium containing 50 mM maltose at 30°C, and then treated with or without 50 mM GlcNAc. Cells were collected at 30 min following treatment for Western and qRT-PCR analysis. Expression level of *NAG1* was normalized with *CDC28*, and mean data ± SD from three independent qRT-PCR experiments was plotted.

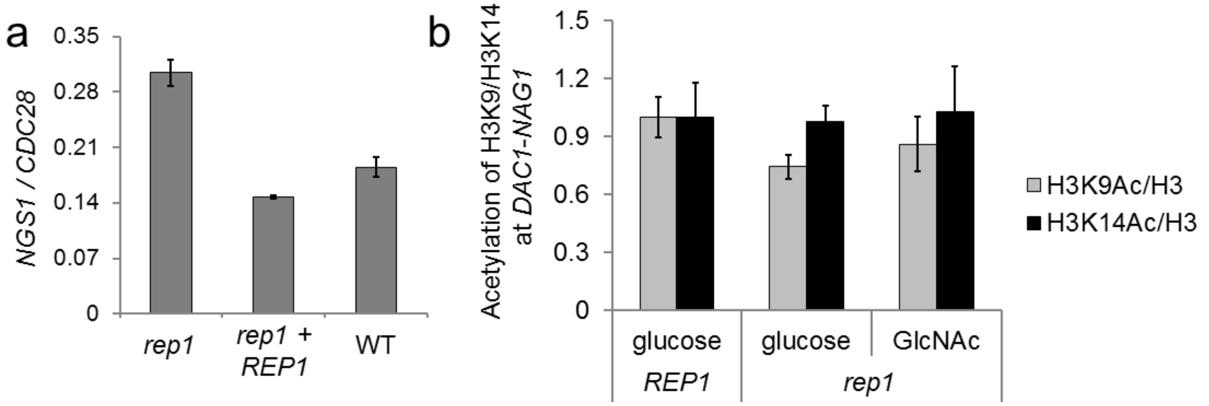

Supplementary Figure 6. The effect of *REP1* deletion on *NGS1* expression and GlcNAc-induced H3K9ac and H3K14ac at the *NAG1-DAC1* promoter. (**a**) Expression level of *NGS1* in *rep1Δ/rep1Δ* mutant (YLO141), *rep1Δ/rep1Δ::REP1* (YLO142), and wild-type (SC5314) cells upon GlcNAc induction. Cells were grown in liquid SC medium with 2.5 mM GlcNAc for 2 h at 30°C for RNA extraction. The signals obtained from *CDC28* mRNA were used for normalization. Mean data ± SD from three independent qRT-PCR experiments was plotted. (**b**) Deletion of *REP1* abolished the increase in H3K9 and H3K14 acetylation in response to GlcNAc. ChIP with anti-H3, anti-acetylated H3K9, and anti-acetylated H3K14 antibodies showed relative levels of H3K9 and H3K14 acetylation at the promoter of *NAG1* and *DAC1* in *rep1Δ/rep1Δ* mutant (YLO141) and *rep1Δ/rep1Δ::REP1* (YLO142) cells. Cells were incubated in SC medium with GlcNAc or glucose at 30°C and collected at 30 min. ChIP DNA was quantitated by qPCR with primers at the promoter region of *NAG1* and *DAC1*. The *ACT1* promoter region was used as a control. The value in *rep1Δ/rep1Δ::REP1* (YLO142) in glucose-containing medium was set to 1.00. The ChIP data showed the average of three independent qPCR data with error bars representing the SD.

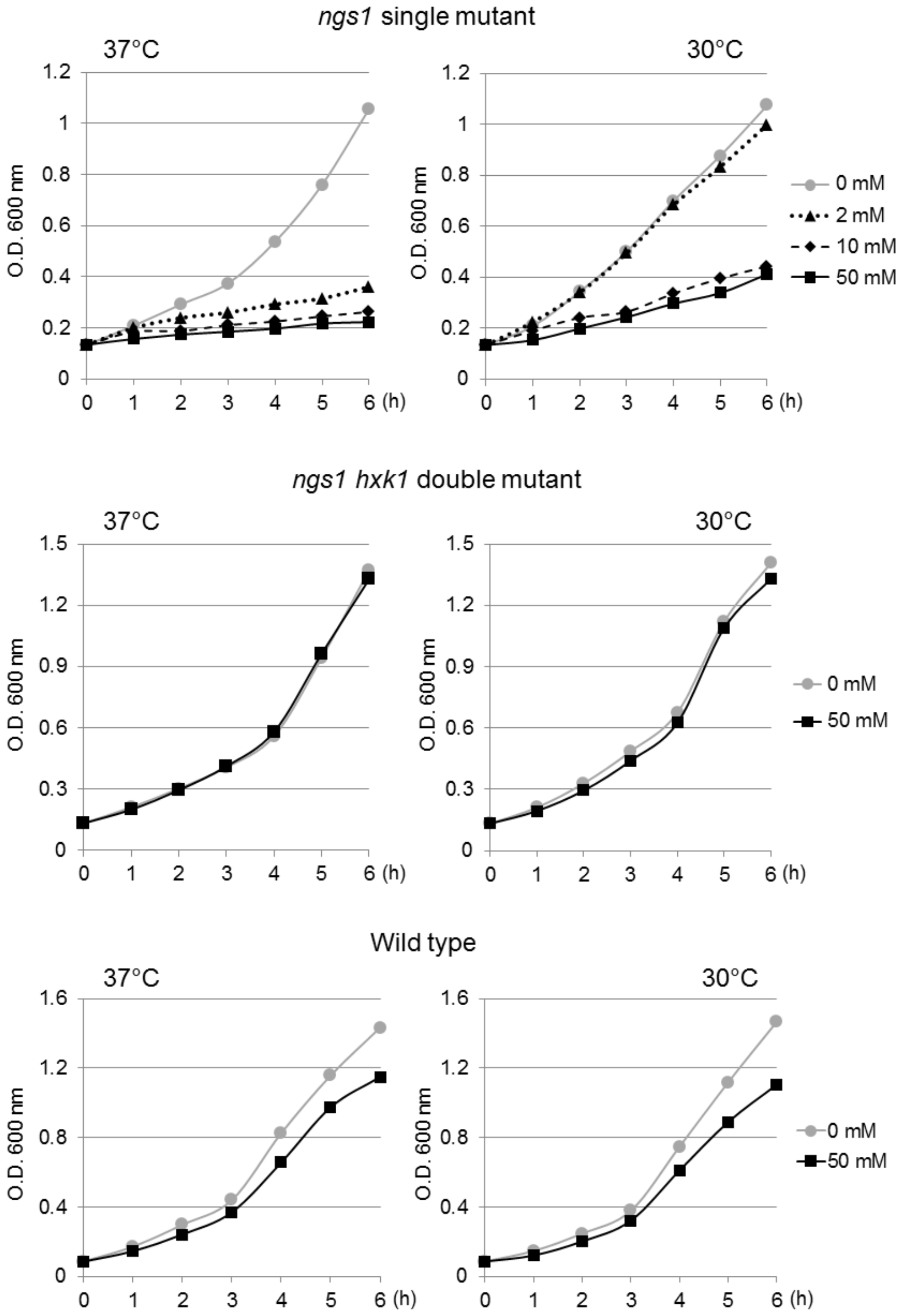

Supplementary Figure 7. The growth inhibitory effect of GlcNAc in *ngs1* cells could be abolished by deleting *HXK1.* Short term growth assays for cells of *ngs1* single mutant (HLY 4391), *ngs1 hxk1* double mutant (HLY4427), and wild type (SC5314) incubated at 30°C or 37°C in SC medium containing 50 mM galactose plus different concentrations of GlcNAc as indicated. Cells were treated with or without GlcNAc after growing overnight at 30°C to early log phase in SC medium plus galactose and then tested for growth at 30°C or 37°C.

Fig. 2c

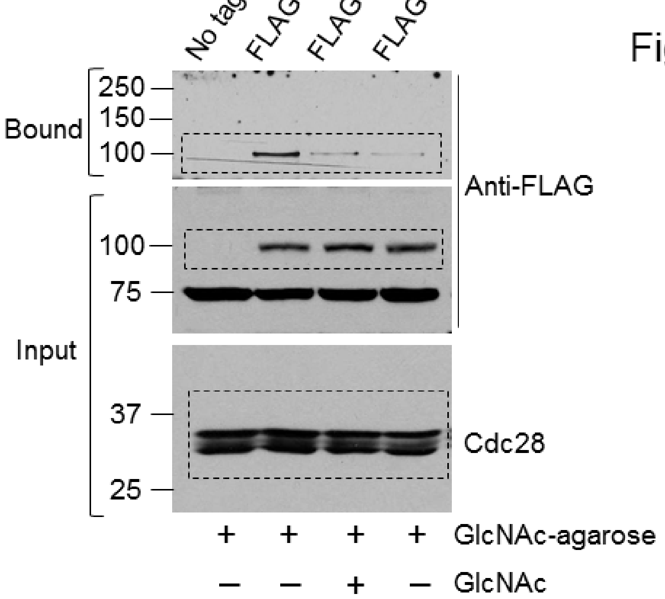

Fig. 4e

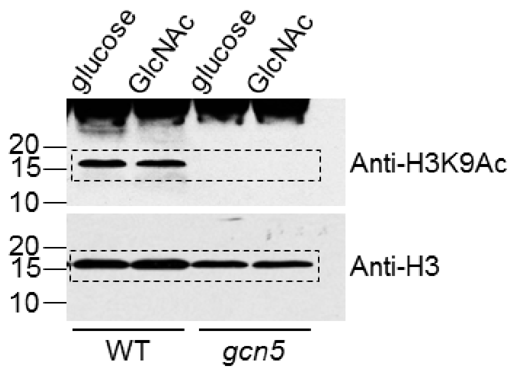

Fig. 4f

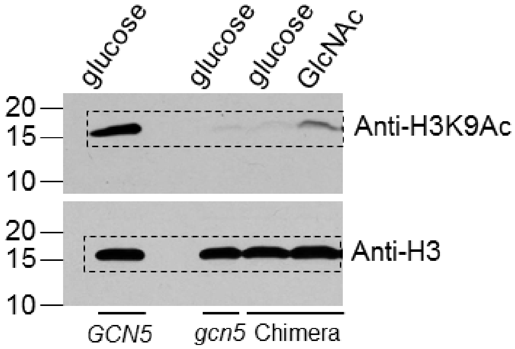

Fig. 6d

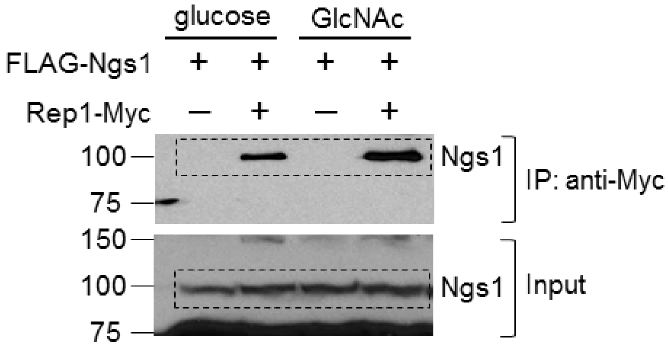

Supplementary Figure 8. Uncropped, full-size scans of Western Blots. Blots correspond to those shown in Figure 2c, Figure 4e, Figure 4f, and Figure 6d of the main manuscript.

Supplementary Table 1. Primers used in this study.

Primer	Sequence (5'-3')[a]	Purpose and features
1	ATAAGAATGCGGCCGCTGCCACAATAGATATGGCAAGTAG	pBES116-NGS1
2	AAAACTGCAGTCACAATCCAAACTTACTCTCTAC	
3	CACCTTCCTCAGCTATGGCAAACATGATTGGATACTTGTAC	pBES116-NGS1^{D74A}
4	GTTTGCCATAGCTGAGGAAGGTGGGATGATGAACTCGTTG	
5	CAATACCTGGGAAAGCAGCCCCCACGGTAAACAACCCTCCATC	pBES116-NGS1$^{KH175/176AA}$
6	TACCGTGGGGGCTGCTTTCCCAGGTATTGGAAACGCTACTGTTG	
7	GTTCCAAAACTAGCAACTCCCACCAACTGATGTGATAGTTTTGTC	pBES116-NGS1^{R145A}
8	TGGTGGGAGTTGCTAGTTTTGGAACCACCATTGAAGATGTG	
9	CTAGTAATGAAGCAACAGTAGCGTTTCCAATACCTGGGAAATG	pBES116-NGS1^{D186A}
10	GAAACGCTACTGTTGCTTCATTACTAGAACTACCCATGATG	
11	GATTTTTCAGCCAATATGTACACAAGATGTCCGCAATTTG	pBES116-NGS1^{V686A}
12	GTACATATTGGCTGAAAAATCAAAACGTTTGCAGAGTATTG	
13	CTAGGGCCCCGTCACTCGTTAACTAATAG	pSFS2-NGT1
14	GTACTCGAGTTGATTTGCTAGTTGGGTTG	
15	ATAAGAATGCGGCCGCATGCTGAGTTAGATGTAGTC	
16	TCCCCGCGGGTAGTAGATGAGACAAGAAC	
17	AGCTTGTCTCATGGTTGATG	*NAG1 and DAC1* promoter
18	GCTTAGGGAGAAAGAACAAC	
19	GTAAGTTTTTAGCAATACTCTGCAAACGTTTTGATTTTTCAAC	pBES116-NGS1^{G696A}
20	CAGAGTATTGCTAAAAACTTACACCTCAGAGCAATACGATATTTG	
21	GTAAGTTTTTTAAAATACTCTGCAAACGTTTTGATTTTTCAAC	pBES116-NGS1^{G696L}
22	CAGAGTATTTTAAAAAACTTACACCTCAGAGCAATACGATATTTG	
23	AAAACTGCAGTCTTCAACTGTTACTTAGGGTTTG	pBES116-NGS1p
24	AAAACTGCAGATG**GACTACAAAGACCATGACGGTGATTATAAAGATCATGACATCGATTACAAGGATGACGATGACAAG**TCCACATTTTATGCTGGACAATTATTATGTG[b]	3×FLAG fusion
25	CTGATCGATCTTCAACCCAACTAGCAAATC	pBA1-NGT1
26	CTAGGTACCCAACAGGATAGATTAGCCAAC	
27	GGCGACGCGTTCCACATTTTATGCTGGAC	MAL2p-GFP-NGS1
28	GGCGACGCGTTCACAATCCAAACTTACTCTCTAC	
29	TGGATTAGCTCGAGCATTTG	*CDC28*-qRTPCR
30	CCAACAGACCACATATCTACCC	
31	AAAACTGCAGTCAGAAAAGTTGTGTCCAGCTCGG	pBES116-NGS1N
32	AAAACTGCAGATGCCTGAAGGTGAAGTATCAGCC	pBES116-NGS1C
33	CTAGGTACCTCACAATCCAAACTTACTCTCTAC	
34	GTTAGTTTGTTCCCCCATACTATTATGACTGAGACTAGCATTAGTGGGTTGCGTGGCCCCAAGTCCATG**TGTGGAATTGTGAGCGGATA**[c]	*HXK1* disruption
35	GTTACTCCAATTACACTAGTGCGATGTACACTTGGGCTTGCTCATTCATCTTATATCATTTTATACATTC**TTTCCCAGTCACGACGTT**[c]	
36	GTAGTGTGTGCACTGCC	*ACT1* promoter
37	CCACCGTCCATTTTGAATGA	
38	AAAACTGCAGATGACAAAATTTTATGCTGGTC	pBES116-CtNGS1
39	CTGGATATCCTAAACGTCTTTTCTTCC	
40	CAATCCCCGGGAAAGCAGCTCCAACAGTAAACAAACCCGCATC	pBES116-CtNGS1$^{KH175/176AA}$
41	GTTTACTGTTGGAGCTGCTTTCCCGGGGATTGGGAATGCTGCTG	
42	CAAACATTTTAAAATGTTTTGACAACGCTTAGACTTGTCAAC	pBES116-CtNGS1^{G700L}
43	CAAAACATTTTAAAATGTTTGCATTCTCGAGCTATGAAATATTTC	

44	AAAACTGCAGATG**GACTACAAAGACCATGACGGTGATTATAAAGATCATGACATCGATTACAAGGATGACGATGACAAG**CCTGAAGGTGAAGTATCAGCCAAGTTACCAC[b]	FLAG-NGS1C
45	CGGGATCCCATGTTGAGTGCAAAATCAGTG	pPR671-chimera-SAT1
46	CTTGATGTACCCCATCATGACACTCTTCTCCGTAGAAACTTGTGTG	
47	GAGAAGAGTGTCATGATGGGGTACATCAAGGATTACGAAGGTG	
48	GGCGACGCGTCGCTATACAAAACTACAGTCTTTC	
49	CGGGATCCCGAGCCACACCCACAATGGATTC	pPR671-REP1-SAT1 pPR673-REP1
50	GGCGACGCGTCGTTTGAAATAAACATTGGAACTC	
51	CGCTACTGTTGACTCATTACTAG	*NGS1*-qRTPCR
52	CAGCAGCACTTATTCCATCTAAC	

[a] Restriction sites are underlined.
[b] Boldface sequences in primers 24 and 44 are 3×FLAG.
[c] Boldface sequences in primers 34 and 35 are segments that anneal to plasmids pGEM-URA3 and pRS-ARG4ΔSpeI for amplification of disruption cassettes.

Supplementary Table 2. *C. albicans* strains used in this study.

Strain	Genotype	Source
SN250	*his1Δ/his1Δ, leu2Δ::C.d. HIS1/leu2Δ::C.m. LEU2, arg4Δ/arg4Δ, URA3/ura3Δ::imm*^{434}*, IRO1/iro1Δ::imm*^{434}	(1)
BWP17	*ura3Δ::imm*^{434}*/ura3Δ::imm*^{434}*, his1Δ::hisG/his1Δ::hisG, arg4Δ::hisG/arg4Δ::hisG*	(2)
SC5314	Wild type	(3)
ngs1 orf19.7516	*his1Δ/his1Δ, leu2Δ/leu2Δ, arg4Δ/arg4Δ, URA3/ura3Δ::imm*^{434}*, IRO1/iro1Δ::imm*^{434}*, ngs1Δ::C.d. HIS1/ngs1Δ::C.m. LEU2*	(1)
nag1	*his1Δ/his1Δ, leu2Δ/leu2Δ, arg4Δ/arg4Δ, URA3/ura3Δ::imm*^{434}*, IRO1/iro1Δ::imm*^{434}*, nag1Δ::C.d. HIS1/nag1Δ::C.m. LEU2*	(1)
snf4	*his1Δ/his1Δ, leu2Δ/leu2Δ, arg4Δ/arg4Δ, URA3/ura3Δ::imm*^{434}*, IRO1/iro1Δ::imm*^{434}*, snf4Δ::C.d. HIS1/snf4Δ::C.m. LEU2*	(1)
HLY4394	*ura3Δ::imm*^{434}*/ura3Δ::imm*^{434}*, his1Δ::hisG/his1Δ::hisG, arg4Δ::hisG/arg4Δ::hisG ngt1Δ::FRT/ngt1Δ::FRT, ADE2/ade2Δ::URA3, RP10/rp10Δ::HIS1*	This study
HLY4391	*his1Δ/his1Δ, leu2Δ/leu2Δ, ARG4/arg4Δ, URA3/ura3Δ::imm*^{434}*, IRO1/iro1Δ::imm*^{434}*, ngs1Δ::C.d. HIS1/ngs1Δ::C.m. LEU2*	This study
HLY4392	*his1Δ/his1Δ, leu2Δ/leu2Δ, ARG4/arg4Δ, ura3Δ/ura3Δ::imm*^{434}*, IRO1/iro1Δ::imm*^{434}*, ngs1Δ::C.d. HIS1/ngs1Δ::C.m. LEU2*	This study
HLY4393	*his1Δ/his1Δ, leu2Δ/leu2Δ, ARG4/arg4Δ, ura3Δ/ura3Δ::imm*^{434}*, IRO1/iro1Δ::imm*^{434}*, ngs1Δ::C.d. HIS1/ngs1Δ::C.m. LEU2, ADE2/ade2Δ::ADH1p-NGT1-URA3*	This study
HLY4395	*his1Δ/his1Δ, leu2Δ/leu2Δ, ARG4/arg4Δ, ura3Δ/ura3Δ::imm*^{434}*, IRO1/iro1Δ::imm*^{434}*, ngs1Δ::C.d. HIS1/ngs1Δ::C.m. LEU2, ADE2/ade2Δ::NGS1p-NGS1-URA3*	This study
HLY4396	*his1Δ/his1Δ, leu2Δ/leu2Δ, ARG4/arg4Δ, ura3Δ/ura3Δ::imm*^{434}*, IRO1/iro1Δ::imm*^{434}*, ngs1Δ::C.d. HIS1/ngs1Δ::C.m. LEU2, ADE2/ade2Δ::URA3*	This study
HLY4397	*his1Δ/his1Δ, leu2Δ/leu2Δ, ARG4/arg4Δ, ura3Δ/ura3Δ::imm*^{434}*, IRO1/iro1Δ::imm*^{434}*, ngs1Δ::C.d. HIS1/ngs1Δ::C.m. LEU2, ADE2/ade2Δ::MAL2p-GFP-NGS1-URA3*	This study
HLY4398	*his1Δ/his1Δ, leu2Δ/leu2Δ, ARG4/arg4Δ, ura3Δ/ura3Δ::imm*^{434}*, IRO1/iro1Δ::imm*^{434}*, ngs1Δ::C.d. HIS1/ngs1Δ::C.m. LEU2, ADE2/ade2Δ::NGS1p-NGS1*^{D74A}*-URA3*	This study
HLY4399	*his1Δ/his1Δ, leu2Δ/leu2Δ, ARG4/arg4Δ, ura3Δ/ura3Δ::imm*^{434}*, IRO1/iro1Δ::imm*^{434}*, ngs1Δ::C.d. HIS1/ngs1Δ::C.m. LEU2, ADE2/ade2Δ::NGS1p-NGS1*^{R145A}*-URA3*	This study
HLY4400	*his1Δ/his1Δ, leu2Δ/leu2Δ, ARG4/arg4Δ, ura3Δ/ura3Δ::imm*^{434}*, IRO1/iro1Δ::imm*^{434}*, ngs1Δ::C.d. HIS1/ngs1Δ::C.m. LEU2, ADE2/ade2Δ::NGS1p-NGS1*$^{KH175/6AA}$*-URA3*	This study
HLY4401	*his1Δ/his1Δ, leu2Δ/leu2Δ, ARG4/arg4Δ, ura3Δ/ura3Δ::imm*^{434}*, IRO1/iro1Δ::imm*^{434}*, ngs1Δ::C.d. HIS1/ngs1Δ::C.m. LEU2, ADE2/ade2Δ::NGS1p-NGS1*^{D186A}*-URA3*	This study
HLY4402	*his1Δ/his1Δ, leu2Δ/leu2Δ, ARG4/arg4Δ, ura3Δ/ura3Δ::imm*^{434}*, IRO1/iro1Δ::imm*^{434}*, ngs1Δ::C.d. HIS1/ngs1Δ::C.m. LEU2, ADE2/ade2Δ::NGS1p-3FLAG-NGS1-URA3*	This study
HLY4403	*his1Δ/his1Δ, leu2Δ/leu2Δ, ARG4/arg4Δ, ura3Δ/ura3Δ::imm*^{434}*, IRO1/iro1Δ::imm*^{434}*, ngs1Δ::C.d. HIS1/ngs1Δ::C.m. LEU2, ADE2/ade2Δ::NGS1p-3FLAG-NGS1*^{D74A}*-URA3*	This study

HLY4404	his1Δ/his1Δ, leu2Δ/leu2Δ, ARG4/arg4Δ, ura3Δ/ura3Δ::imm^{434}, IRO1/iro1Δ::imm^{434}, ngs1Δ::C.d. HIS1/ngs1Δ::C.m. LEU2, ADE2/ade2Δ::NGS1p-3FLAG-NGS1^{R145A}-URA3	This study
HLY4405	his1Δ/his1Δ, leu2Δ/leu2Δ, ARG4/arg4Δ, ura3Δ/ura3Δ::imm^{434}, IRO1/iro1Δ::imm^{434}, ngs1Δ::C.d. HIS1/ngs1Δ::C.m. LEU2, ADE2/ade2Δ::NGS1p-3FLAG-NGS1$^{KH175/6AA}$-URA3	This study
HLY4406	his1Δ/his1Δ, leu2Δ/leu2Δ, ARG4/arg4Δ, ura3Δ/ura3Δ::imm^{434}, IRO1/iro1Δ::imm^{434}, ngs1Δ::C.d. HIS1/ngs1Δ::C.m. LEU2, ADE2/ade2Δ::NGS1p-NGS1^{V686A}-URA3	This study
HLY4407	his1Δ/his1Δ, leu2Δ/leu2Δ, ARG4/arg4Δ, ura3Δ/ura3Δ::imm^{434}, IRO1/iro1Δ::imm^{434}, ngs1Δ::C.d. HIS1/ngs1Δ::C.m. LEU2, ADE2/ade2Δ::NGS1p-NGS1^{G696A}-URA3	This study
HLY4408	his1Δ/his1Δ, leu2Δ/leu2Δ, ARG4/arg4Δ, ura3Δ/ura3Δ::imm^{434}, IRO1/iro1Δ::imm^{434}, ngs1Δ::C.d. HIS1/ngs1Δ::C.m. LEU2, ADE2/ade2Δ::NGS1p-NGS1^{G696L}-URA3	This study
HLY4427	hisΔ1/his1Δ, leu2Δ/ leu2Δ, arg4Δ/arg4Δ, ura3Δ/ura3Δ::imm^{434}, IRO1/iro1Δ::imm^{434}, ngs1Δ::C.d. HIS1/ngs1Δ::C.m. LEU2, hxk1Δ::URA3/hxk1Δ::ARG4	This study
CPS50	ura3::imm434::URA3/ura3::imm434 iro1::IRO1/iro1::imm434 his1::hisG/his1::hisG leu2/leu2 arg4/arg4 gcn5::PLP/gcn5::PHP	(4)
YLO141	ura3Δ::imm^{434}/ura3Δ::imm^{434}, his1Δ::hisG/his1Δ::hisG, arg4Δ::hisG/arg4Δ::hisG rep1Δ::ARG4/rep1Δ::URA3-dpl200 ENO1/eno1::ENO1-tetR-ScHAP4-3xHA-HIS	(5)
YLO142	ura3Δ::imm^{434}/ura3Δ::imm^{434}, his1Δ::hisG/his1Δ::hisG, arg4Δ::hisG/arg4Δ::hisG rep1Δ::ARG4/rep1Δ::URA3-dpl200-REP1-HIS1	(5)
CAI4	ura3Δ::imm^{434}/ura3Δ::imm^{434}	(3)
HLY4451	ura3Δ::imm^{434}/ura3Δ::imm^{434}, his1Δ::hisG/his1Δ::hisG, arg4Δ::hisG/arg4Δ::hisG rep1Δ::ARG4/rep1Δ::ura3Δ ENO1/eno1::ENO1-tetR-ScHAP4-3xHA-HIS	This study
HLY4452	ura3Δ::imm^{434}/ura3Δ::imm^{434}, his1Δ::hisG/his1Δ::hisG, arg4Δ::hisG/arg4Δ::hisG rep1Δ::ARG4/rep1Δ::ura3Δ ENO1/eno1::ENO1-tetR-ScHAP4-3xHA-HIS RP10::ACT1p-REP1-13MYC-URA3	This study
HLY4453	ura3Δ::imm^{434}/ura3Δ::imm^{434}, his1Δ::hisG/his1Δ::hisG, arg4Δ::hisG/arg4Δ::hisG rep1Δ::ARG4/rep1Δ::ura3Δ ENO1/eno1::ENO1-tetR-ScHAP4-3xHA-HIS ADE2/ade2Δ::NGS1p-3FLAG-NGS1-URA3	This study
HLY4454	his1Δ/his1Δ, leu2Δ/leu2Δ, ARG4/arg4Δ, ura3Δ/ura3Δ::imm^{434}, IRO1/iro1Δ::imm^{434}, ngs1Δ::C.d. HIS1/ngs1Δ::C.m. LEU2, ADE2/ade2Δ::NGS1p-3FLAG-NGS1-URA3 RP10::ACT1p-REP1-13MYC-SAT1	This study
HLY4455	ura3::imm434::URA3/ura3::imm434 iro1::IRO1/iro1::imm434 his1::hisG/his1::hisG leu2/leu2 arg4/arg4 gcn5::PLP/gcn5::PHP ADE2/ade2Δ::ADH1p-C.d. ARG4 NGS1/ngs1Δ::NGS1p-Chimera-SAT1	This study
HLY4456	his1Δ/his1Δ, leu2Δ/leu2Δ, ARG4/arg4Δ, ura3Δ/ura3Δ::imm^{434}, IRO1/iro1Δ::imm^{434}, ngs1Δ::C.d. HIS1/ngs1Δ::C.m. LEU2, ADE2/ade2Δ::NGS1p-C.t. NGS1-URA3	This study
HLY4457	his1Δ/his1Δ, leu2Δ/leu2Δ, ARG4/arg4Δ, ura3Δ/ura3Δ::imm^{434}, IRO1/iro1Δ::imm^{434}, ngs1Δ::C.d. HIS1/ngs1Δ::C.m. LEU2, ADE2/ade2Δ::NGS1p-C.t. NGS1$^{KH175/6AA}$-URA3	This study
HLY4458	his1Δ/his1Δ, leu2Δ/leu2Δ, ARG4/arg4Δ, ura3Δ/ura3Δ::imm^{434}, IRO1/iro1Δ::imm^{434}, ngs1Δ::C.d. HIS1/ngs1Δ::C.m. LEU2, ADE2/ade2Δ::NGS1p-C.t. NGS1^{G700L}-URA3	This study

HLY4459	*ura3Δ::imm^434^/ura3Δ::imm^434^, ADE2/ade2Δ::NGS1p-3FLAG-NGS1-URA3*	This study
HLY4460	*his1Δ/his1Δ, leu2Δ/leu2Δ, ARG4/arg4Δ, ura3Δ/ura3Δ::imm^434^, IRO1/iro1Δ::imm^434^, ngs1Δ::C.d. HIS1/ngs1Δ::C.m. LEU2, ADE2/ade2Δ::NGS1p-3FLAG-NGS1N-URA3*	This study
HLY4461	*his1Δ/his1Δ, leu2Δ/leu2Δ, ARG4/arg4Δ, ura3Δ/ura3Δ::imm^434^, IRO1/iro1Δ::imm^434^, ngs1Δ::C.d. HIS1/ngs1Δ::C.m. LEU2, ADE2/ade2Δ::NGS1p-3FLAG-NGS1C-URA3*	This study
HLY4462	*his1Δ/his1Δ, leu2Δ/leu2Δ, ARG4/arg4Δ, ura3Δ/ura3Δ::imm^434^, IRO1/iro1Δ::imm^434^, ngs1Δ::C.d. HIS1/ngs1Δ::C.m. LEU2, ADE2/ade2Δ::NGS1p-3FLAG-NGS1^V686A^-URA3*	This study
HLY4463	*his1Δ/his1Δ, leu2Δ/leu2Δ, ARG4/arg4Δ, ura3Δ/ura3Δ::imm^434^, IRO1/iro1Δ::imm^434^, ngs1Δ::C.d. HIS1/ngs1Δ::C.m. LEU2, ADE2/ade2Δ::NGS1p-3FLAG-NGS1^G696L^-URA3*	This study

1. Noble SM, French S, Kohn LA, Chen V, & Johnson AD (2010) Systematic screens of a Candida albicans homozygous deletion library decouple morphogenetic switching and pathogenicity. *Nature genetics* 42(7):590-598.
2. Wilson RB, Davis D, & Mitchell AP (1999) Rapid hypothesis testing with Candida albicans through gene disruption with short homology regions. *Journal of bacteriology* 181(6):1868-1874.
3. Fonzi WA & Irwin MY (1993) Isogenic strain construction and gene mapping in Candida albicans. *Genetics* 134(3):717-728.
4. Chang P, Fan X, & Chen J (2015) Function and subcellular localization of Gcn5, a histone acetyltransferase in Candida albicans. *Fungal genetics and biology : FG & B* 81:132-141.
5. Chen CG*, et al.* (2009) Rep1p negatively regulating MDR1 efflux pump involved in drug resistance in Candida albicans. *Fungal genetics and biology : FG & B* 46(9):714-720.
